# Phosphorylation of glutaminase by PKCε is essential for its enzymatic activity and critically contributes to tumorigenesis

**DOI:** 10.1038/s41422-018-0021-y

**Published:** 2018-03-07

**Authors:** Tianyu Han, Weihua Zhan, Mingxi Gan, Fanrong Liu, Bentong Yu, Y. Eugene Chin, Jian-Bin Wang

**Affiliations:** 1 Institute of Translational Medicine, Nanchang University, Nanchang City, Jiangxi 330031 China; 2 School of Life Sciences, Nanchang University, Nanchang City, Jiangxi 330031 China; 3 Department of Pathology, The Second Affiliated Hospital of Nanchang University, Nanchang City, Jiangxi 330006 China; 4 Department of Cardiovascular Surgery, The First Affiliated Hospital of Nanchang University, Nanchang City, Jiangxi 330006 China; 5 Institute of Health Sciences, Chinese Academy of Sciences at Shanghai, Shanghai, 200025 China

## Abstract

Glutamine metabolism plays an important role in cancer development and progression. Glutaminase C (GAC), the first enzyme in glutaminolysis, has emerged as an important target for cancer therapy and many studies have focused on the mechanism of enhanced GAC expression in cancer cells. However, little is known about the post-translational modification of GAC. Here, we report that phosphorylation is a crucial post-translational modification of GAC, which is responsible for the higher glutaminase activity in lung tumor tissues and cancer cells. We identify the key Ser314 phosphorylation site on GAC that is regulated by the NF-κB-PKCε axis. Blocking Ser314 phosphorylation by the S314A mutation in lung cancer cells inhibits the glutaminase activity, triggers genetic reprogramming, and alleviates tumor malignancy. Furthermore, we find that a high level of GAC phosphorylation correlates with poor survival rate of lung cancer patients. These findings highlight a previously unappreciated mechanism for activation of GAC by phosphorylation and demonstrate that targeting glutaminase activity can inhibit oncogenic transformation.

## Introduction

Altered cancer cell metabolism has been long recognized as a common event in cancer progression. A hallmark of these alterations is the increased utilization of glucose and secretion of lactate even in the presence of oxygen and is known as the Warburg effect.^1^ Another corresponding alteration is elevated glutamine metabolism.^2^ As the most abundant amino acid in the plasma, glutamine is synthesized in most tissues as a non-essential amino acid, but this can change when cells, particularly tumor cells, have a heavy demand for glutamine that exceeds its supply. Hence, glutamine is referred to as a “conditionally” essential amino acid.^3^ In tumor cells, glutamine can be metabolized to enter the tricarboxylic acid cycle to satisfy bioenergetic demands and macromolecular synthesis.^4,5^ In addition to metabolic needs, glutamine also plays important roles in cell signaling and gene expression.^6,7^

As the initial metabolic enzyme in glutaminolysis, glutaminase catalyzes the conversion of glutamine to glutamate and ammonia. There are two glutaminase isoforms that are encoded by different genes in human cells: the liver-type glutaminase, also known as *LGA* or *GLS2* and the kidney-type glutaminase which is known as *KGA* or *GLS1*.^4^ An upregulation of glutaminase C (GAC), a splice variant of *GLS1*, has been demonstrated in cancer cells in comparison to normal cells.^8–11^ In addition, inhibition of glutaminase activity or glutaminase depletion blocked cancer cell growth.^12^ As the importance of glutamine metabolism in cancer progression, great efforts have been made to target this metabolic pathway for cancer treatment. Both the glutamine analog L-DON and allosteric inhibitors of glutaminase (BPTES and compound 968, respectively) have been intensively studied as cancer therapeutics.^12–14^ Recently, a potent and selective inhibitor of glutaminase, CB-839, has been reported. CB-839 exhibited anti-proliferative activity in triple-negative breast cancer cells but not in estrogen receptor positive tumor cells. CB-839 also promoted a tumor-specific pharmacodynamic response and had in vivo efficacy in breast cancer xenograft models, both as a single agent or in combination with the standardly used agent, paclitaxel. These studies demonstrate that glutaminase is an effective therapeutic target in cancer therapy.^15^

The mechanisms underlying the regulation of GAC activity in cancer cells remained largely unclear. It was reported that c-Myc could promote the proliferation of prostate cancer and B lymphoma cell lines by suppressing miR-23a/b and subsequently increasing the GLS1 expression level.^16^ In colon cancer, a lncRNA called CCAT2 that interacts with the CFIm complex fine-tunes the alternative splicing of glutaminase (*GLS*) by selecting the poly(A) site in intron 14 of the precursor mRNA, resulting in the preferential expression of the GAC isoform.^17^ In breast cancer cells, activated c-jun induced GLS expression by directly binding to the *GLS* promoter region. The expression level of c-jun also correlated positively with the sensitivity of breast cancer cells to treatment with GLS inhibitor.^18^ In our previous study, we found that the high glutaminase activity in breast cancer cells was regulated by Rho GTPases through transcription factor NF-κB.^12^ This was the first report that glutaminase activity, not its expression level, plays a critical role in cancer progression. The role of Rho GTPases in regulating NF-κB has been studied,^19,20^ however, the exact mechanism of NF-κB in regulating glutaminase activity is still not well understood. In non-small cell lung cancer (NSCLC), the mechanism for regulating GAC activity has not yet been studied. Here, we have shown that NSCLC cells exhibit much higher glutaminase activity than normal human bronchial epithelial (HBE) cells and the high glutaminase activity in the cancer cells results from GAC phosphorylation. We identified Serine 314 as the key phosphorylation site in GAC, and PKCε, the responsible kinase, as a new target of NF-κB (p65). We found that highly phosphorylated GAC closely correlates with poor patient survival. Thus, these findings offer a new mechanism for regulating GAC activity in lung cancer cells and shed new light on the therapeutic strategy for NSCLC treatment.

## Results

### Glutaminase C activity is elevated in NSCLC and regulated by phosphorylation

**Fig. 1:**
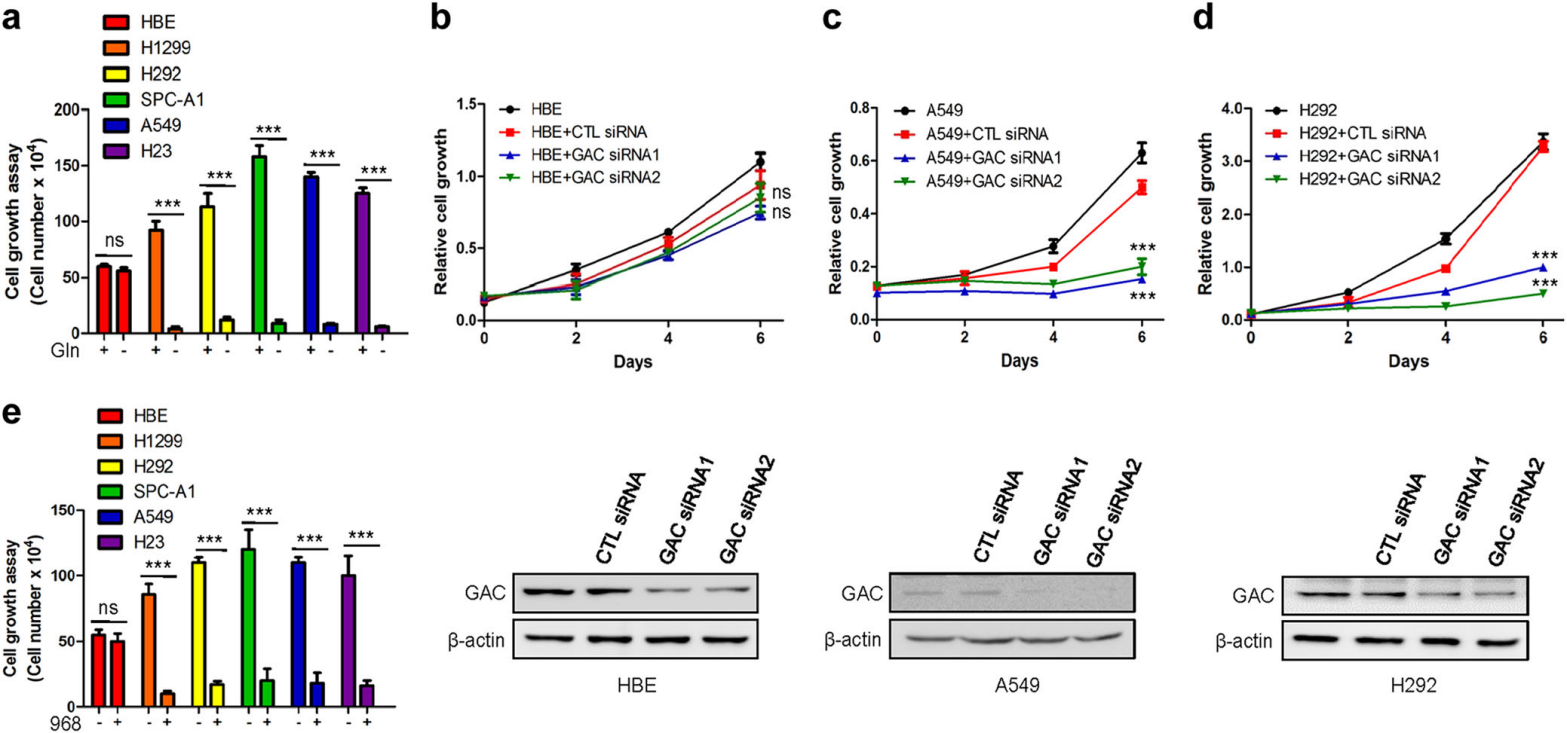
Importance of glutaminase in the growth of NSCLC. **a** NSCLC cells (H1299, H292, SPC-A1, A549, and H23) were cultured in RPMI 1640 with 10% FBS in the presence or absence of glutamine for 6 days; and normal human bronchial epithelial (HBE) cells were cultured in Airway epithelial cell basal medium in the presence or absence of glutamine for 6 days before cells were trypsinized and counted. Data represent the average of three independent experiments (mean ± SD). ***P < 0.001, ns: *P* > 0.05. **b**–**d** NSCLC cells were cultured in RPMI 1640 with 10% FBS and HBE cells were cultured in Airway epithelial cell basal medium, cells were transfected with either control siRNA or GAC siRNAs. At the indicated times, cells were fixed in 3.7% formaldehyde and stained with 0.1% crystal violet. Dye was extracted with 10% acetic acid and the relative proliferation was assessed from the increase in absorbance at 595 nm. Data represent the average of three independent experiments (mean ± SD). ***P < 0.001, ns: *P* > 0.05 (top figures). The knockdown efficiency was determined by western blotting using anti-GAC antibody (bottom figures). **e** NSCLC cell lines (H23, H1299, H292, A549, and SPC-A1) were cultured in RPMI 1640 with 10% FBS in the presence or absence of 10 μM 968 for 6 days; HBE cells were cultured in Airway epithelial cell basal medium in the presence or absence of 10 μM 968 for 6 days, then cells were trypsinized and counted. Data represent the average of three independent experiments (mean ± SD). ***P < 0.001, ns: *P* > 0.05

To determine the importance of glutamine metabolism in NSCLC cells, we used multiple NSCLC cell lines (H23, H1299, H292, A549, and SPC-A1) and normal human bronchial epithelial cells (HBE) as a control in cell growth assays. The cells were cultured in the presence or absence of glutamine. The NSCLC cells proliferated rapidly in normal medium, but their growth was inhibited in glutamine free medium. In contrast, the growth of HBE cells was only slightly decreased in glutamine free medium (Fig. 1a). Thus, the growth of NSCLC cells appears more dependent on glutamine than the growth of HBE cells. We next sought to investigate if the glutamine dependence was related to GAC. When GAC was depleted, this significantly inhibited the growth of NSCLC cells but not HBE cells (Fig. 1b–d and Supplementary information, Figure S1A-C). To further confirm that the reduced growth of NSCLC cells was a consequence of GAC knockdown, we overexpressed exogenous GAC with V5-tag at its C terminus (V5-GAC) in tumor cells depleted for endogenous GAC. We found that by rescuing the expression of GAC, we could recover the reduced growth of H1299 cells resulting from GAC knockdown (Figure S1D). Similar inhibitory effects on the growth of NSCLC cells were also observed following treatment with our previously reported GAC-specific inhibitor, 968 (Fig. 1e).^12^ These data suggest that GAC plays a crucial role in NSCLC development and progression.

**Fig. 2:**
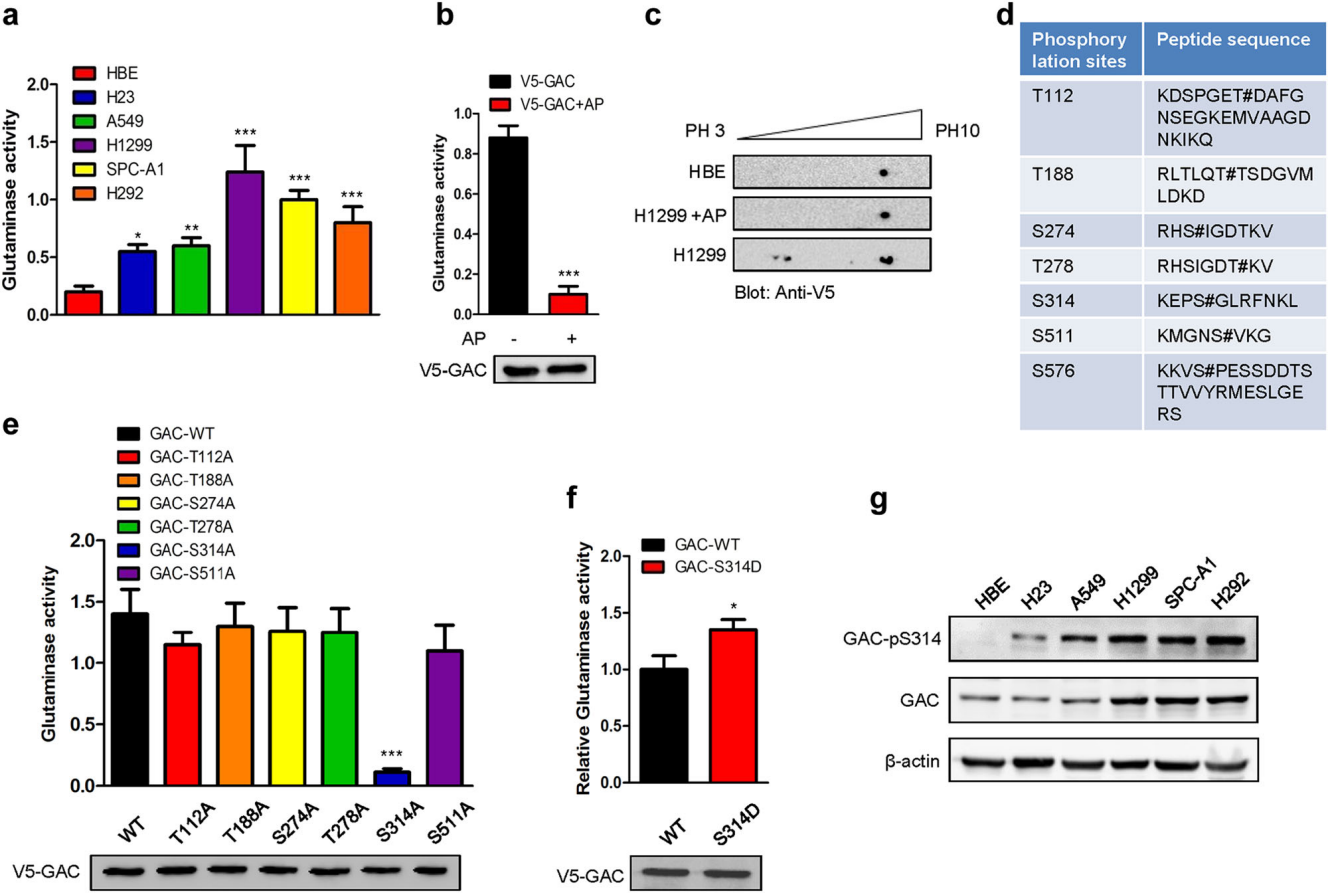
Glutaminase C activity is elevated in NSCLC and regulated by phosphorylation. **a** Mitochondrial fractions were isolated from equivalent numbers of NSCLC cells and HBE cells, and then the glutaminase activity was determined. Data represent the average of three independent experiments (mean ± SD). *P < 0.05, **P < 0.01, ***P < 0.001. **b** H1299 cells were transiently transfected with pcDNA3.1-V5-GAC and then immunoprecipitated using an anti-V5 antibody. The samples were treated with alkaline phosphatase or left untreated for 1 h at 37 °C. Glutaminase activity was then assayed. Data represent the average of three independent experiments (mean ± SD). ***P < 0.001 (AP alkaline phosphatase) (top figure). The GAC expression levels were checked by western blotting using an anti-V5 antibody (bottom figure). **c** HBE cells transiently transfected with V5-GAC were lysed and extracts subjected to isoelectric focusing; H1299 cells transiently transfected with V5-GAC were lysed and subjected to alkaline phosphatase or control treatment prior to isoelectric focusing. All the above experiments were followed by western blotting using an anti-V5 antibody. **d** The GAC phosphorylation sites in H1299 cells but not in HBE cells were identified by mass spectrometry. # represents a phosphorylation site. **e** H1299 cells transiently transfected with V5-GAC wild-type (GAC-WT) or GAC mutants (T112A, T188A, S274A, T278A, S314A, and S511A), were immunoprecipated using the anti-V5 antibody and then the glutaminase assay was performed. Data represent the average of three independent experiments (mean ± SD). ***P < 0.001 (top figure). The GAC expression levels of wild-type and mutants were checked by western blotting with the anti-V5 antibody (bottom figure). **f** H1299 cells transiently transfected with V5-GAC wild-type (GAC-WT) or GAC mutant (GAC-S314D) were immunoprecipated using the anti-V5 antibody before glutaminase assay. Data represent the average of three independent experiments (mean ± SD). *P < 0.05 (top figure). The GAC expression levels of wild-type and mutant were checked by western blotting using the anti-V5 antibody (bottom figure). **g** The expression of total and phosphorylated GAC at serine 314 in NSCLC cell lines and HBE cells was determined by western blotting using anti-GAC and anti-GAC-pS314 antibodies

Since NSCLC cells showed a stronger dependence on glutamine than normal HBE cells, we investigated if this dependence correlated with GAC expression levels. We first examined the mRNA expression levels of GAC in HBE and NSCLC cells. Most NSCLC cells exhibited a higher mRNA level of GAC than HBE cells (Supplementary information, Figure S2A). We subsequently assessed the protein levels of GAC in these cells and found levels in A549 and H23 cells were similar to HBE cells (Supplementary information, Figure S2B). When we examined the glutaminase activity, we found it was much higher in NSCLC cells than in HBE cells (Fig. 2a). We then turned to examine GAC expression in tumor tissues and adjacent normal tissues from 10 NSCLC patients. We found that the expression levels of GAC and glutaminase activity were higher in most tumor tissues than in normal lung tissues (Supplementary information, Figure S2C, D). Taken together, this indicates that GAC activity is elevated in NSCLC.

Our previous studies indicated that GAC activity might be regulated by phosphorylation in breast cancer cells.^12^ To investigate whether a similar molecular mechanism is responsible for the elevated GAC activity in NSCLC cells, we expressed V5-GAC in H1299 cells and immunoprecipitated GAC using an anti-V5 antibody to determine the effects of alkaline phosphatase treatment on glutaminase activity. The alkaline phosphatase-treated V5-GAC displayed reduced glutaminase activity compared with untreated V5-GAC (Fig. 2b), suggesting that phosphorylation plays an important role in regulating GAC activity in NSCLC cells. To confirm this result, we carried out isoelectric focusing to separate the phosphorylated forms of GAC. In HBE cells, the GAC appeared as a single spot. However, in H1299 cells, we observed multiple spots, several of which were shifted toward pH 3.0. When the same samples were treated with alkaline phosphatase, shifting of the spots was diminished (Fig. 2c). This suggested that the different levels of glutaminase activity between NSCLC and HBE cells might be due to different phosphorylation levels. We therefore immunoprecipitated endogenous GAC from H1299 or HBE cells by GAC antibody and undertook mass spectrometric analysis. Ten phosphorylation sites were identified in GAC immunoprecipitated from H1299 cells while only six phosphorylation sites were detected in HBE cells (Supplementary information, Table S1). We just focused on the phosphorylation sites which differed in H1299 cells from those in HBE cells, namely, Thr112, Thr188, Ser274, Thr278, Ser314, Ser511, and Ser576 (Fig. 2d). We mutated each of these residues into alanines, transfected the mutants into H1299 cells, and assessed glutaminase activity. Mutation of serine 314 to alanine (S314A) but not the other phosphorylation sites drastically reduced glutaminase activity (Fig. 2e). In contrast, GAC mutant with Ser314 changed to aspartic acid (S314D) showed higher activity than the wild-type GAC (Fig. 2f). We then generated a rabbit polyclonal antibody recognizing an epitope including phosphorylated Ser314 (verified by western blot, Supplementary information, Figure S3A, B). We used this antibody to examine the phosphorylation levels of endogenous GAC at Ser314 (GAC-pS314) in HBE and NSCLC cells. Whereas the signal was almost undetectable in HBE cells, the extent of phosphorylation of GAC at Ser314 in A549, SPC-A1, H1299, and H292 cells was quite obvious (Fig. 2g). To determine whether this regulatory mechanism is generally found in other cancer cell types, we treated lysates of different cancer cells with alkaline phosphatase, and then assessed glutaminase activity. This revealed that alkaline phosphatase treatment remarkably reduced glutaminase activity in lung cancer cells, hepatocarcinoma cells, breast cancer cells, and cervical cancer cells. In addition, the phosphorylation level of GAC at Ser314 was also downregulated (Supplementary information, Figure S3C-H). Together, these results demonstrate that regulation of GAC activity by phosphorylation is a general phenomenon in various tumor types. We then assessed the effects of GAC (S314A) on the proliferation of NSCLC cells. The growth rate of all cell lines overexpressing GAC (S314A) was significantly slower than cells overexpressing wild-type GAC (Supplementary information, Figure S4A-C). These findings suggest that the phosphorylation of GAC at Ser314 plays a key role in the regulation of glutaminase activity and the growth of NSCLC cells.

### PKCε is responsible for the phosphorylation of GAC at Ser314

**Fig. 3:**
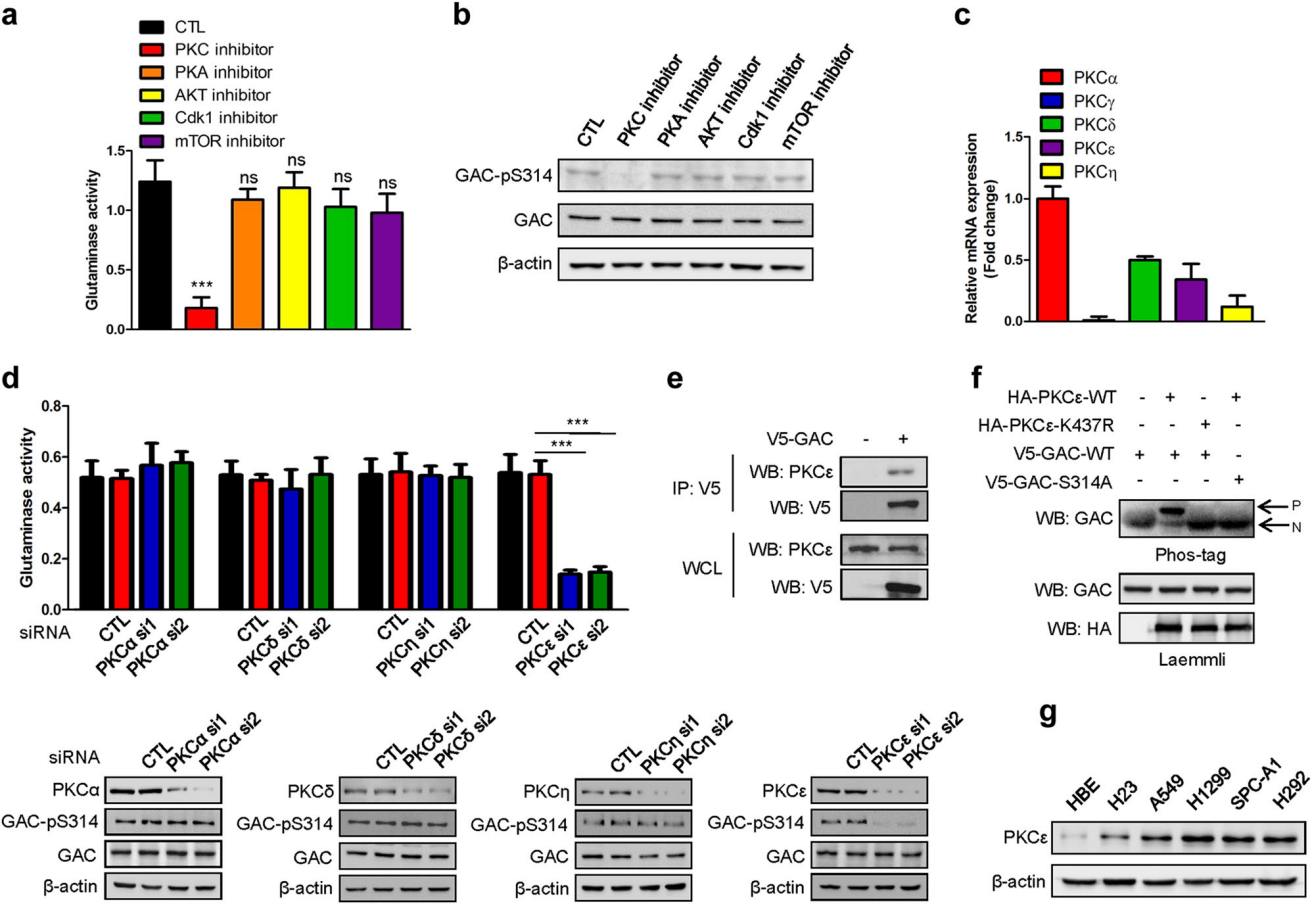
PKCε is the kinase that phosphorylates GAC at Ser314. **a** H1299 cells were pretreated with the generic PKC inhibitor (Staurosporine, 50 nM), PKA inhibitor (H89, 20 μM), AKT inhibitor (LY294002, 10 μM), CDK1 inhibitor (Roscovitine, 15 μM), and mTOR inhibitor (Rapamycin, 100 nM) for 24 h. Mitochondria were then isolated and then the glutaminase activity was detected. Data represent the average of three independent experiments (mean ± SD). ***P < 0.001, ns: *P* > 0.05. **b** Lysates from the above experiments were collected and the phospho-GAC and total GAC expression levels were determined by western blotting using anti-GAC-pS314 and anti-GAC antibodies. **c** The mRNA levels of different PKC isoforms were determined by q-PCR in H1299 cells. Data represent the average of three independent experiments (mean ± SD). **d** H1299 cells were transiently transfected with siRNAs targeting PKCα, PKCδ, PKCε, and PKCη separately; mitochondrial fractions were isolated and then the glutaminase activity was detected. Data represent the average of three independent experiments (mean ± SD). ***P < 0.001 (top figure). The knockdown efficiency of the siRNAs targeting PKCα, PKCδ, PKCε, and PKCη, the expressions of total GAC and phosphorylated GAC were determined by western blotting using the indicated antibodies (bottom figures, CTL control siRNA). **e** H1299 cells transfected with or without V5-GAC were lysed for immunoprecipitation using an anti-V5 antibody and blotted with indicated antibodies. **f** HBE cells separately transfected with the indicated plasmids were lysed for immunoprecipitation using an anti-HA or an anti-V5 antibody. In vitro kinase assays were performed and analyzed using Phos-tag and Laemmli SDS-PAGE, followed by immunoblotting using the indicated antibodies (P: phosphoprotein, N: non-phosphorylated protein). **g** Expression levels of PKCε in NSCLC cells and HBE cells were determined by western blotting using an anti-PKCε antibody

To determine which kinase can phosphorylate GAC and thus affect the GAC activity, we used the Netphosk program to predict the possible kinases for GAC phosphorylation. PKA, PKC, and Cdk1 were found to be the likely candidates. As in addition, AKT has been reported to influence glycolytic metabolism^21,22^ and since the mTORC1 pathway was demonstrated to regulate glutamine metabolism,^23^ we used inhibitors directed against PKA, PKC, Cdk1, AKT, and mTOR to treat H1299 cells and determined whether glutaminase was affected. We found that inhibition of PKC, but none of the other kinases largely abrogated GAC activity and the phosphorylation of GAC at Ser314 (Fig. 3a, b). Similar inhibitory effects were also observed in hepatocarcinoma cells, breast cancer cells, and cervical cancer cells (Supplementary information, Figure S5A-F). These results indicate that PKC is the major kinase responsible for GAC phosphorylation at Ser314 and that this is a general phenomenon in many tumor types and not just NSCLC. To test the efficacy of each inhibitors used in these experiments, we assessed the phosphorylation state of known target molecules: CREB for PKA; pro-caspase-3 for PKC; PCNA for CDK1; Akt for Akt; and eIF4EBP1 for mTOR and found each inhibitor to be effective (Figure S6). As there are ten PKC isoforms: PKCα, PKCβI, PKCβII, PKCγ, PKCδ, PKCε, PKCζ, PKCη, PKCθ, and PKCι/λ,^24^ we sought to identify which PKC isoform plays a key role in regulating GAC phosphorylation and activity. We first considered the isoforms targeted by the PKC inhibitor we had used, staurosporine. These are PKCα, PKCδ, PKC**ε**, PKCγ, and PKCη. We carried out q-PCR to check their mRNA expression levels in H1299 cells and eliminated PKCγ as its expression level was too low to be considered as a potential candidate (Fig. 3c). We therefore used RNAi to deplete the PKCα, PKCδ, PKCε, and PKCη isoforms in H1299 cells and then assessed glutaminase activity. Only the knockdown of PKCε significantly inhibited GAC activity and reduced levels of GAC-pS314 (Fig. 3d). Co-immunoprecipitation experiment showed that PKCε could interact with GAC (Fig. 3e). To further demonstrate that PKCε could phosphorylate GAC, we performed in vitro kinase assays using Phos-tag, a dinuclear metal complex that acts as a selective phosphate-binding tag molecule, to enhance the electrophoretic mobility shift. HBE cells were transfected separately with plasmids expressing HA-PKCε-WT, HA-PKCε-(K437R), V5-GAC-WT, and V5-GAC-S314A, the individual proteins were immunoprecipitated from cell extracts, and in vitro kinase assays were performed (Fig. 3f). PKCε induced a significantly shifted band above the primary band of GAC, indicating the phosphorylation of GAC by PKCε. The dominant-negative counterpart, PKCε-K437R, did not elucidate this effect. Moreover, the phosphorylation of GAC by PKCε was abolished by mutating serine 314 to alanine. These results demonstrate that PKCε is able to phosphorylate GAC at Ser314. Accordingly, we found that expression of PKCε in HBE cells is low, whereas it is actively expressed in H23, A549, SPC-A1, H1299, and H292 cells (Fig. 3g). This result is consistent with the observation of differential phosphorylation of GAC at S314 in NSCLC and HBE cells (Fig. 2g).

### The NF-κB-PKCε axis is crucial for the regulation of GAC activity and the growth of NSCLC cells

**Fig. 4:**
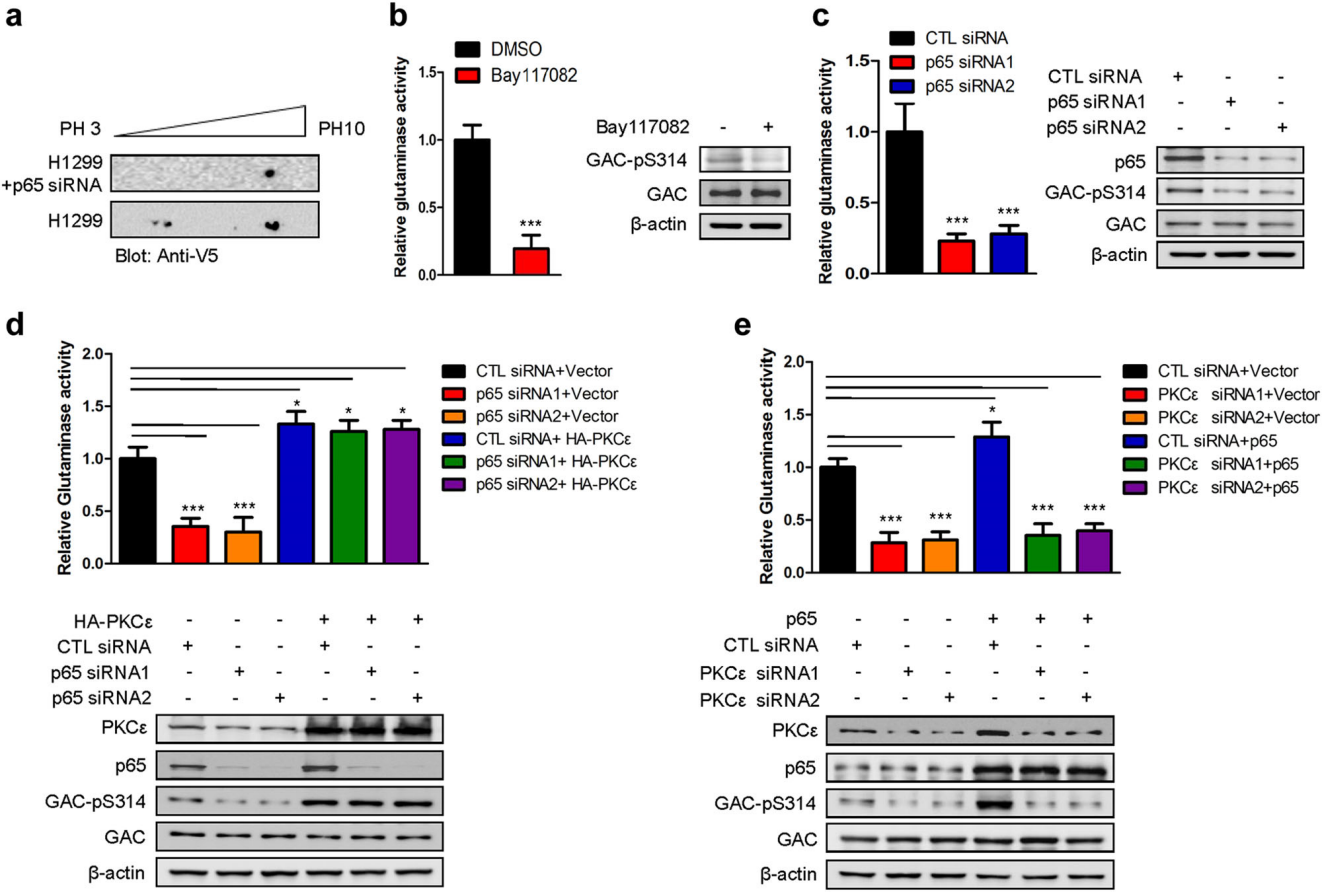
The activity of GAC is regulated by the NF-κB-PKCε axis. **a** H1299 cells transfected with V5-GAC, and NF-κB (p65) siRNA or controls were lysed and subjected to isoelectric focusing followed by western blotting using anti-V5 antibody. **b** Mitochondria were isolated from H1299 cells treated or untreated with Bay117082 and then the glutaminase activity was detected. Data represent the average of three independent experiments (mean ± SD). ***P < 0.001 (left figure). Protein expression was determined by western blotting using indicated antibodies (right figure). **c** H1299 cells were transfected with control siRNA (CTL siRNA) or NF-κB (p65) siRNAs and then mitochondria were isolated, and the glutaminase activity was detected. Data represent the average of three independent experiments (mean ± SD). ***P < 0.001 (left figure). Protein expression was probed using the indicated antibodies (right figure). **d** H1299 cells were co-transfected with control siRNA (CTL siRNA) or p65 siRNAs and empty vector or pCMV-HA-PKCε before mitochondria were isolated and the glutaminase activity was detected. Data represent the average of three independent experiments (mean ± SD). *P < 0.05, ***P < 0.001 (top figure). Protein expression was probed using indicated antibodies (bottom figure). **e** H1299 cells were co-transfected with control siRNA (CTL siRNA) or PKCε siRNAs and empty vector or pcDNA3.0-p65 before mitochondria were isolated, and the glutaminase activity was detected. Data represent the average of three independent experiments (mean ± SD). *P < 0.05, ***P < 0.001 (top figure). Protein expression was probed using indicated antibodies (bottom figure)

In our previous work, we reported that GAC activity in breast cancer cells can be regulated by NF-κB.^12^ To determine whether the GAC activity in NSCLC cells can also be regulated by NF-κB, we performed another isoelectric focusing experiment and found mobility-shifted phosphorylated spots were diminished when NF-κB (p65) was depleted from H1299 cells (Fig. 4a). This result indicated that GAC activity was likely modulated through phosphorylation in an NF-κB-dependent manner. To validate this hypothesis, we measured glutaminase activity and found that the enzymatic activity was reduced significantly when cells were treated with the NF-κB inhibitor, Bay117082 (Fig. 4b, left panel). The phosphorylation of GAC at Ser314 was also reduced following this treatment (Fig. 4b, right panel). Reduction of glutaminase activity and the phosphorylation level of GAC at Ser314 were also observed when p65 was knocked down in H1299 and A549 cells (Fig. 4c and Supplementary information, Figure S7A). However, mutant GAC (S314D) was resistant to the inhibitory effects of either Bay117082 treatment or knockdown of p65 (Supplementary information, Figure S7B, C). These results demonstrated that NF-κB regulates GAC activity by affecting the phosphorylation of GAC at Ser314. We next wanted to determine whether the effect of NF-κB on GAC activity was mediated by PKCε. We found that p65 knockdown reduced both GAC activity and the extent of phosphorylation of GAC at Ser314, whereas overexpression of PKCε rescued the expression of GAC-pS314 and GAC activity resulting from p65 knockdown (Fig. 4d). However, the overexpression of p65 could not restore reduced GAC-pS314 and GAC activity caused by PKCε knockdown (Fig. 4e). Thus, NF-κB (p65) appears to mediate regulation of GAC activity through PKCε.

**Fig. 5:**
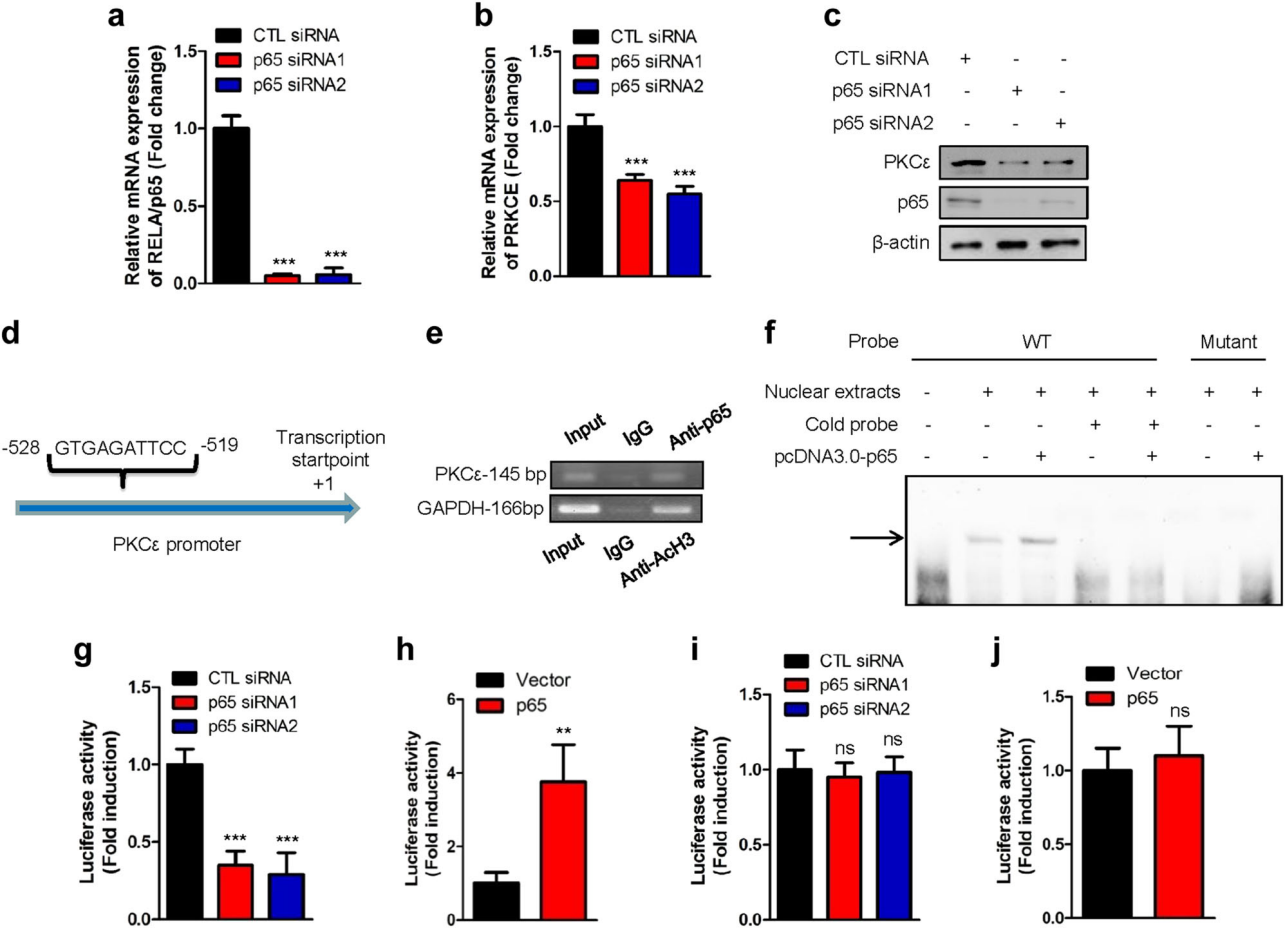
PKCε is a new downstream target of NF-κB. **a**,** b** H1299 cells were transiently transfected with p65 siRNAs. Forty-eight hours later, total RNAs were extracted. The mRNA levels of p65 (**a**) and PKCε (**b**) were determined by q-PCR. Data represent the average of three independent experiments (mean ± SD). ***P < 0.001. **c** H1299 cells were transiently transfected with p65 siRNAs. Forty-eight hours later, the cells were lysed. Protein expression was assessed by western blotting using the indicated antibodies. **d** Schematic depiction of the NF-κB binding sequence in the promoter region of PKCε. **e** ChIP assay was conducted with anti-NF-κB (p65), anti-AcH3 antibodies, or control rabbit IgG for immunoprecipitation, followed by PCR with PKCε promoter-specific and GAPDH promotor-specific primers. **f** H1299 cells were transfected with or without pcDNA3.0-p65 plasmid. Nuclear proteins were extracted and subjected to EMSA assay using biotin-labeled probes containing the NF-κB binding site. The arrow indicates probes that bound to NF-κB (p65). **g** pGL3-enhancer vector containing PKCε promoter fragment was transfected into H1299 cells, co-transfected with control or NF-κB (p65) siRNAs and Renilla control plasmid. The relative levels of luciferase activity were normalized to the levels of untreated cells and to the levels of luciferase activity of the Renilla control plasmid. Data represent the average of three independent experiments (mean ± SD). ***P < 0.001. **h** pGL3-enhancer vector containing PKCε promoter fragment was transfected into H1299, co-transfected with Renilla control plasmid and pcDNA3.0 vector or pcDNA3.0-p65 plasmid. The relative levels of luciferase activity were normalized to the levels of vector control and to the levels of luciferase activity of the Renilla control plasmid. Data represent the average of three independent experiments (mean ± SD). **P < 0.01. **i** pGL3-enhancer vector containing PKCε promoter fragment with mutations in NF-κB binding site was transfected into H1299 cells, co-transfected with control or NF-κB (p65) siRNAs and Renilla control plasmid. The relative levels of luciferase activity were normalized to the levels of untreated cells and to the levels of luciferase activity of the Renilla control plasmid. Data represent the average of three independent experiments (mean ± SD). ns: *P* > 0.05. **j** pGL3-enhancer vector containing PKCε promoter fragment with mutations in NF-κB binding site was transfected into H1299, co-transfected with Renilla control plasmid and pcDNA3.0 vector or pcDNA3.0-p65 plasmid. The relative levels of luciferase activity were normalized to the levels of vector control and to the levels of luciferase activity of the Renilla control plasmid. Data represent the average of three independent experiments (mean ± SD). ns: *P* > 0.05

We then addressed whether PKCε could be regulated by NF-κB. We therefore knocked down p65 and found that both the mRNA and protein levels of PKCε were affected (Fig. 5a–c). Analysis of the promoter region of the PKCε gene identified a putative NF-κB binding sequence (GTGAGATTCC) (Fig. 5d). Chromatin immunoprecipitation showed that p65 could bind to the PKCε promoter (Fig. 5e). The electrophoretic mobility shift assay also showed that p65 could bind to the biotin-labeled probes containing an NF-κB binding site but not probes with mutation in the binding site (Fig. 5f). We therefore introduced the promoter region of PKCε into the pGL3-enhancer vector and performed luciferase assays. We found that the activity of the PKCε promoter decreased dramatically when p65 was knocked down (Fig. 5g, and Supplementary information, Figure S8A) but increased when p65 was overexpressed (Fig. 5h, and Supplementary information, Figure S8B) as shown by luciferase activity assay in H1299 and A549 cells. When we mutated the p65 binding motif in the PKCε promoter (GTTAGATGTT), the luciferase activity did not change irrespective of whether NF-κB (p65) was overexpressed or depleted (Fig. 5i, j). These results showed that PKCε is a new downstream target of NF-κB (p65). These experiments also revealed that luciferase activity driven by PKCε promoter was much higher in H1299 cells than in HBE cells (Supplementary information, Figure S8C). These data highlight a new molecular mechanism by which GAC activity is regulated by the NF-κB-PKCε axis in NSCLC cells.

We then examined the effects of NF-κB (p65) on the growth of NSCLC cells. Treatment with Bay117082 significantly reduced the proliferation of H1299 cells (Supplementary information, Figure S9A). Knocking down p65 using specific siRNAs also drastically inhibited the proliferation rate of NSCLC cells (Supplementary information, Figure S9B, C). Similar results were also obtained by foci formation assay in H1299 cells (Supplementary information, Figure S9D). We then investigated the effects of p65 on glutamine metabolism and found that knockdown of p65 significantly reduced the production of glutamate in H1299 cells (Supplementary information, Figure S9E). We also observed accumulation of glutamine in such p65-depleted H1299 cells (Supplementary information, Figure S9F). Many studies have indicated that PKCε is an oncogene. In accord with this idea, we found that the proliferation and ability of NSCLC cells to form foci were inhibited following PKCε knockdown (Supplementary information, Figure S10A-E). To further determine the biological roles of PKCε in regulating glutamine metabolism, we depleted PKCε in H1299 cells and examined the production of glutamine and glutamate. This revealed that glutamine accumulated while the glutamate production was reduced in H1299 cells following PKCε knockdown (Supplementary information, Figure S10F, G). These results demonstrate that the NF-κB-PKCε axis affects proliferation of NSCLC cells.

### S314A mutation in endogenous *GLS* gene promotes lung differentiation events in A549 cells

**Fig. 6:**
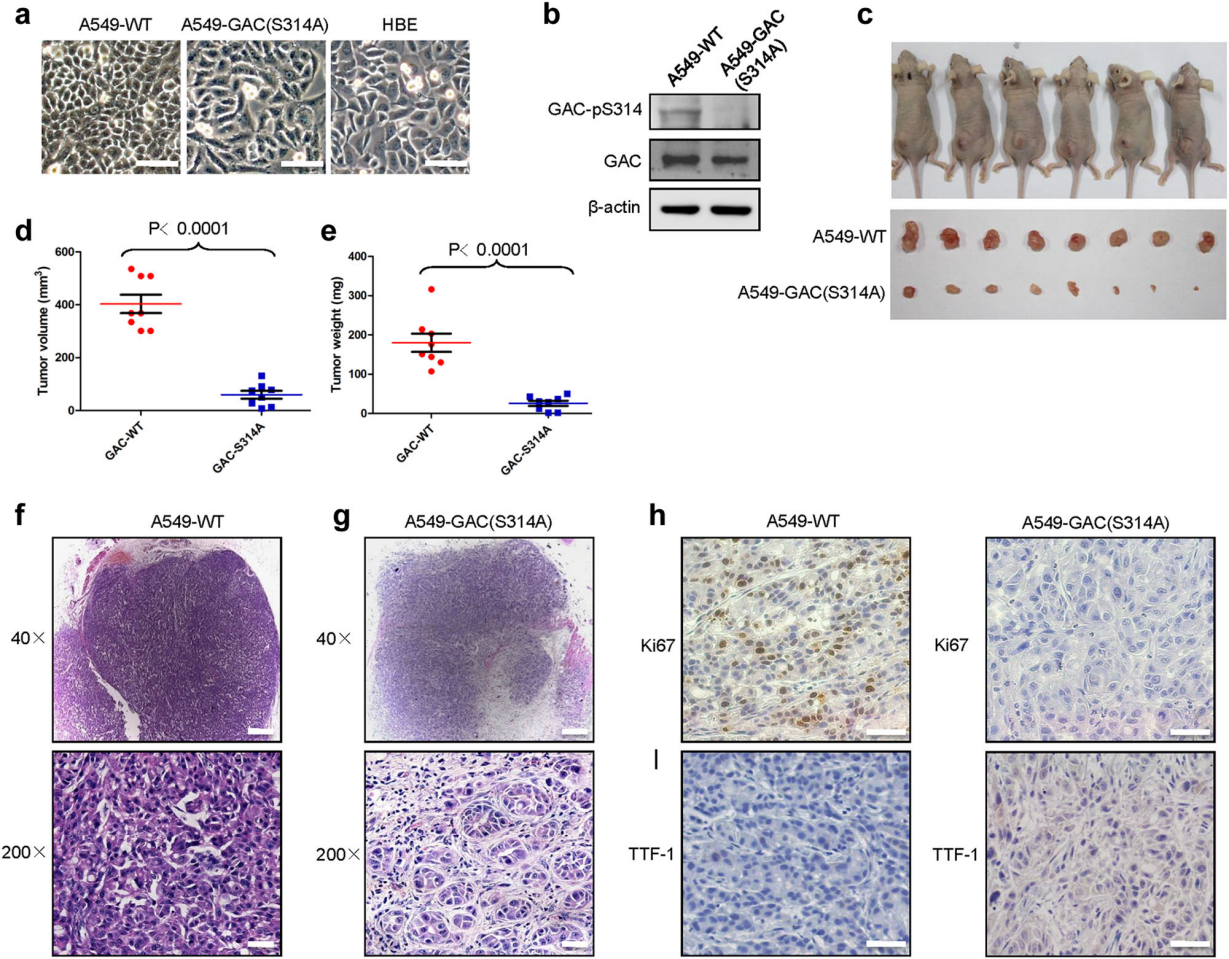
Mutation of serine 314 to alanine in the endogenous *GLS* gene in A549 cells induces lung cancer differentiation. **a** Appearance of HBE, parental A549, and A549-GAC (S314A) cells after being cultured in Airway epithelial cell basal medium and RPMI 1640 with 10% FBS medium separately. Scale bars: 100 μm. **b** The expression of indicated proteins in parental A549 and A549-GAC (S314A) was determined by western blotting using the indicated antibodies. **c**–**e** Parental A549 cells and A549-GAC (S314A) mutant cells (1 × 10^7^) were subcutaneously injected into the flanks of nude mice. Four weeks later, tumors were dissected out, photographed (**c**), and their weights and volumes measured. The *p* value was calculated by paired *t *test (**d**, **e**). **f**,** g** Photomicrographs of hematoxylin-eosin (HE) staining for tumors induced by parental A549 cells (**f**) and A549-GAC (S314A) cells (**g**). Magnification of upper figures is ×40, Scale bars: 200 μm. The magnification of lower figures is ×200, Scale bars: 20 μm. **h**,** i** Immunohistochemical staining in tumors induced by parental A549 cells and A549-GAC(S314A) cells for Ki67 (**h**) and TTF1 (**i**). Magnification is ×200, Scale bars: 50 μm

We next wished to assess the effects of mutating Ser314 of the endogenous *GLS* gene to an alanine residue and so generated such a mutation by CRISPR/Cas9 mutagenesis of A549 cells, A549-GAC (S314A) (Supplementary information, Figure S11A). The resulting cells exhibited some morphological changes compared with parental A549 cells and had a similar morphology to HBE cells (Fig. 6a). When we searched for phosphorylation of GAC at Ser314 in A549-GAC (S314A) cells using the anti-GAC-pS314 antibody, we were unable to detect phosphorylated GAC. However, the expression of total GAC was similar to that in A549-WT cells (Fig. 6b). We found that in contrast to the transformed phenotypes of the parental A549 cells, the A549-GAC (S314A) cells were unable to form colonies in soft agar (Supplementary information, Figure S11B) and showed slower cell proliferation rates (Supplementary information, Figure S11C). Moreover, the A549-GAC (S314A) cells were more susceptible to 968 treatment than A549-WT cells (Supplementary information, Figure S11D). This raised the possibility that combined inhibition of GAC phosphorylation and enzyme activity could be a promising therapeutic strategy for NSCLC. We then assessed the tumorigenetic ability of A549-GAC (S314A) cells in a xenograft model. We observed that xenografts of A549-GAC (S314A) cells displayed a dramatically reduced tumor size (by approximately 95–99%) and weight (by 70–98%) compared with parental A549 cells (Fig. 6c–e). HE staining showed that the tumors arising from the parental A549 cells were heterogeneous in cell size and had many giant cells, and cells with disordered nucleo-cytoplasmic ratios, and pathological fission, indicative of very poor differentiation (Fig. 6f). However, the structure of the adenoid gland could still be easily observed in tumors formed by A549-GAC (S314A) cells. Moreover, tumor cells derived from A549-GAC (S314A) were much more uniform in size with morphologies suggestive of a high degree of differentiation (Fig. 6g). To confirm these results, we detected expression of genes indicative of the differentiation states of lung adenocarcinoma by immunohistochemistry. Ki67 is widely used as a proliferation marker in pathological assessments.^25^ We found that the tumors formed by parental A549 cells were Ki67-positive, whereas tumors derived from A549-GAC(S314A) cells were Ki67-negative (Fig. 6h). NK2 homeobox 1, also known as thyroid TF-1 (TTF-1), was demonstrated to be frequently suppressed in high-grade lung adenocarcinoma.^26^ The tumors formed by A549-WT cells were TTF1-negative, whereas TTF1-positive cells were observed in tumors formed by A549-GAC (S314A) cells (Fig. 6i). These results indicate that the inhibition of GAC phosphorylation at serine 314 promotes lung cancer differentiation.

**Fig. 7:**
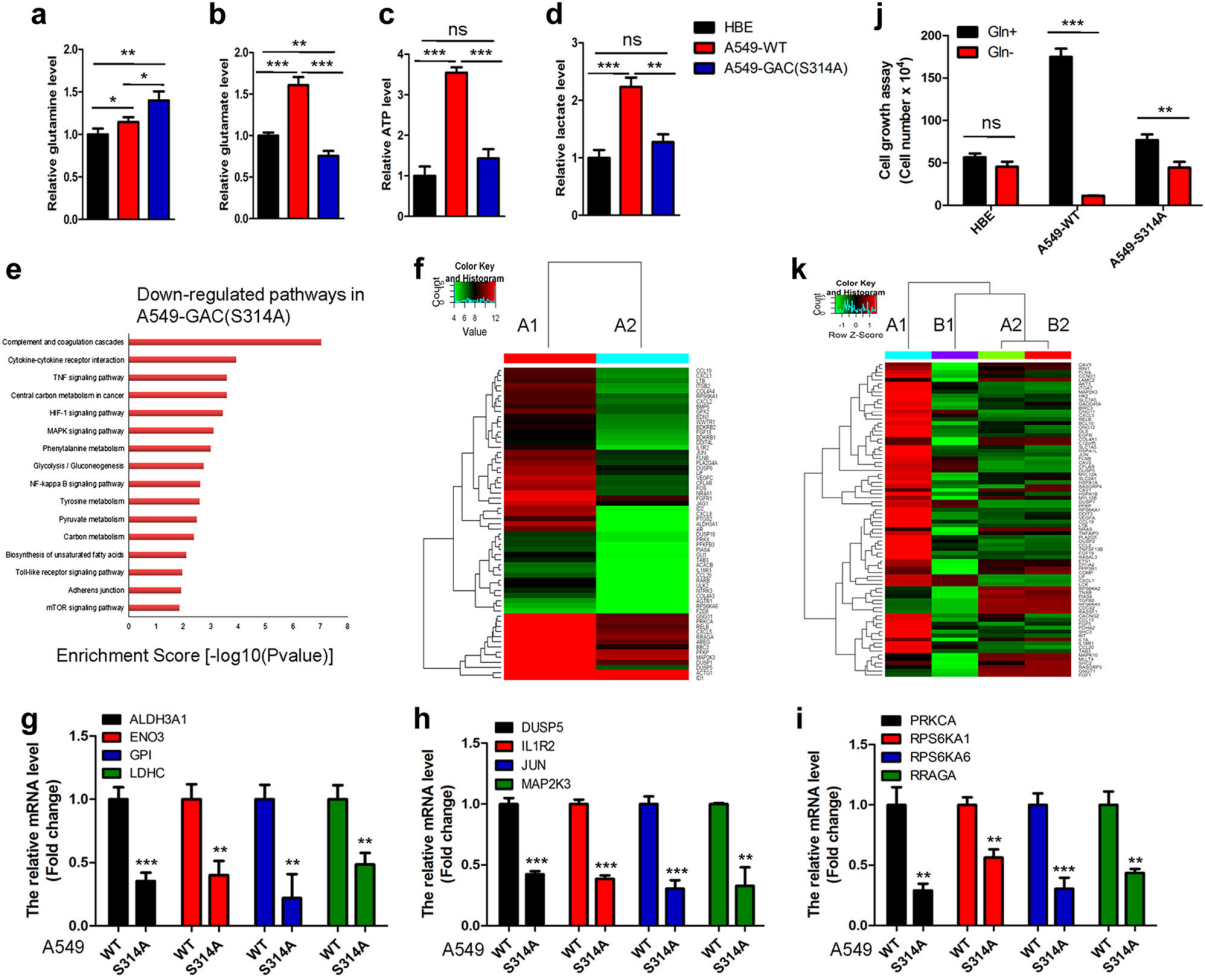
Inhibition of glutamine metabolism induces metabolic reprogramming and reverses transformed phenotypes. **a**–**d** HBE, A549-WT, and A549-GAC (S314A) cells were cultured and metabolites including glutamine (**a**), glutamate (**b**), ATP (**c**), and lactate (**d**) were determined by kits. *P < 0.05, **P < 0.01, ***^/^*P* < 0.001, ns: *P* > 0.05. **e** Signaling pathways downregulated in A549-GAC (S314A) cells compared with parental A549 cells are shown. **f** Heat map of Agilent whole human genome oligo microarray data demonstrates differences in gene expression profiles between parental A549 cells (A1) and A549-GAC (S314A) cells (A2). **g**–**i** Genes participating in glycolysis (**g**), MAPK signaling (**h**), and mTOR signaling (**i**) were analyzed by q-PCR. Data represent the average of three independent experiments (mean ± SD). **P < 0.01, ***P < 0.001. **j** Cell growth assay. HBE cells were cultured in Airway epithelial cell basal medium in the presence or absence of glutamine for 6 days; parental A549 and A549-GAC(S314A) cells were cultured in RPMI 1640 containing 10% FBS in the presence or absence of glutamine for 6 days, then cells were trypsinized and counted. Data represent the average of three independent experiments (mean ± SD). **P < 0.01, ***P < 0.001, ns: *P* > 0.05. **k** Heat map of Agilent whole human genome oligo microarray data, which demonstrated the differences in gene expression profiles among A549 cells cultured in glutamine added medium (A1), in glutamine free medium (B1); A549-GAC(S314A) cells cultured in glutamine added medium (A2) and in glutamine free medium (B2)

To determine the metabolic changes responsible for different phenotypes between parental A549 and A549-GAC (S314A) cells, we assessed the concentrations of metabolites in these two cell types. Glutamine, the substrate of glutaminase, accumulated significantly in A549-GAC(S314A) cells compared with parental A549 cells (Fig. 7a), whereas the concentrations of metabolites such as glutamate (Fig. 7b), ATP (Fig. 7c), and lactate (Fig. 7d) decreased in A549-GAC(S314A) cells to levels similar to those in HBE cells. We then performed metabolomics analysis to compare the concentrations of different metabolites in parental A549 and A549-GAC (S314A) cells (Supplementary information, Table S2 and Figure S12). This revealed that the production of lactate was higher in the parental A549 cells than in A549-GAC (S314A) cells, indicative of enhanced glycolysis. The production of glutamate was reduced and glutamine accumulated in A549-GAC (S314A) cells (Supplementary information, Figure S12), in accord with our previous results (Fig. 7a, b, d). We also observed that production of metabolites of the TCA cycle including succinate and fumarate were higher in the parental A549 cells than in A549-GAC (S314A) cells. There was also significant accumulation of NAD^+^ in A549-GAC (S314A) cells (Supplementary information, Figure S12). As we know, the TCA cycle is the center of metabolism and the main pathway for the production of NADH. Our findings indicate that the TCA cycle is severely impaired in A549-GAC (S314A) cells and further demonstrate that glutamine metabolism could be used to influence the TCA cycle as previously described. Moreover, we found that the UMP and IMP, precursors for the synthesis of nucleotides, accumulated significantly in A549-GAC (S314A) cells (Supplementary information, Figure S12), suggesting impairment of DNA synthesis. This could explain the reduced proliferation rate of A549-GAC (S314A) cells.

To further understand the mechanism underlying reduced tumorigenesis of A549-GAC (S314A) cells, we performed an Agilent whole human genome oligo microarray assay to compare global gene expression profiles in parental A549 and A549-GAC (S314A) cells. Examination of genes exhibiting greater than a twofold difference in expression levels coupled to pathway analysis (Supplementary information, Tables S3–5) identified a series of pathways playing crucial roles in cancer initiation and progression to be significantly downregulated in A549-GAC(S314A) cells (Fig. 7e). The significantly altered pathways included the glycolysis pathway, the HIF-1 pathway, the MAPK pathway, the NF-κB pathway, and the mTOR pathway. These findings were in agreement with previously published microarray data comparing HBE and A549 cells.^27^ We selected 61 genes that participated in these cancer-associated pathways for cluster mapping on the MeV microarray analysis platform. As can be seen from the heat map, these genes were significantly downregulated in A549-GAC (S314A) cells (Fig. 7f). We next selected 12 genes that participated in the glycolysis pathway (Fig. 7g), the MAPK signaling pathway (Fig. 7h), and the mTOR pathway (Fig. 7i) for real-time PCR to confirm the microarray results. We found that these genes were significantly downregulated in A549-GAC (S314A) cells compared with the parental A549 cells.

We already found that proliferation of HBE cells showed much less dependence on glutamine than the proliferation of NSCLC cells (Fig. 1a). To examine if A549-GAC (S314A) cells exhibit similar characteristics to HBE cells, we performed a cell growth assay in medium with or without glutamine. We found that the growth of A549-GAC (S314A) cells was slightly affected by glutamine starvation compared with the parental A549 cell line (Fig. 7j). We also investigated the global gene expression profile by microarray analysis of cells grown in glutamine free medium. When we considered genes showing differential expression of greater than twofold, we found that 1773 genes were downregulated and 2679 genes were upregulated in parental A549 cells grown in glutamine free medium (Supplementary information, Tables S6 and 7). However, there were only 204 genes downregulated and 138 genes upregulated when A549-GAC (S314A) cells were grown in glutamine free medium (Supplementary information, Tables S8 and 9). Pathway analysis indicated that a series of pathways involved in cell proliferation and migration (including the MAPK pathway, the NF-κB pathway, and the Ras pathway) were strongly perturbed in parental A549 cells cultured in glutamine free medium, but not in A549-GAC(S314A) cells cultured in glutamine free medium (Supplementary information, Table S10). We next selected 82 genes involved in cell proliferation and migration for cluster mapping. It was clear that these genes were downregulated significantly when parental A549 cells were grown in glutamine free medium. Conversely, few changes were seen in A549-GAC (S314A) cells (Fig. 7k). Taken together, these results indicate that blocking glutamine metabolism promotes lung cancer cell differentiation and significantly inhibits lung cancer initiation and progression.

### GAC phosphorylation is upregulated in NSCLC and correlates to poor survival of NSCLC patients

**Fig. 8:**
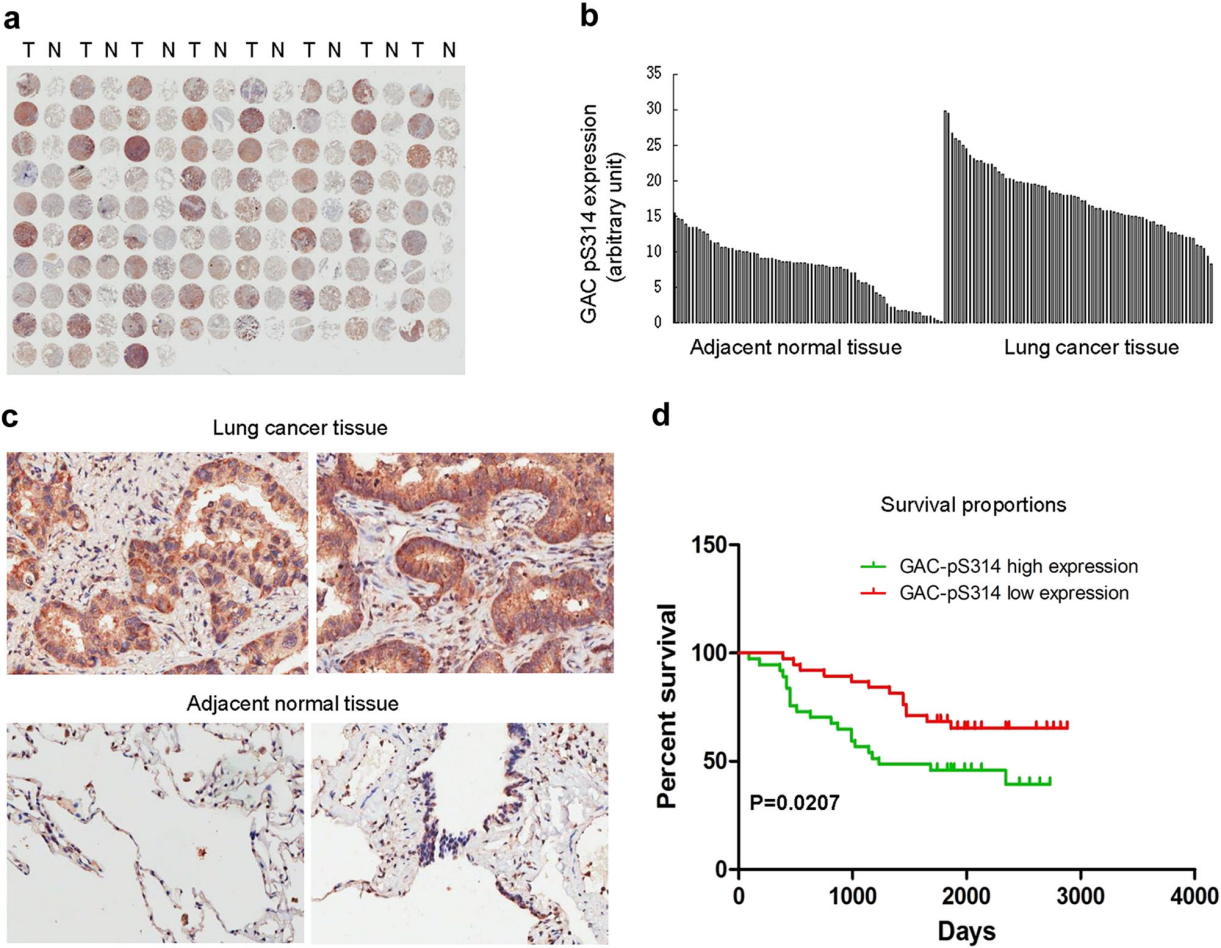
GAC phosphorylation is upregulated in NSCLC and closely related to the poor survival of NSCLC patients. **a** Immunohistochemical staining of a representative lung adenocarcinoma tissue microarray with an anti-GAC-pS314 antibody. T, tumor tissue; N, adjacent normal tissue. **b** Quantification of the immunohistochemical (IHC) staining shown in Fig. 8a. **c** Microscopic evaluation of IHC staining of two representative tumor tissues and adjacent normal tissues shown in Fig. 8a with an anti-GAC-pS314 antibody (brown) and haematoxylin counterstain (blue). **d** Kaplan–Meier survival curve of 75 NSCLC patients. Patients were divided into two groups according to the average staining density of GAC-pS314 in cancer tissues of the tissue array (high expression: *n* = 37, low expression: *n* = 38, Log-rank (Mantel–Cox) test was used for the statistical analysis)

To further determine whether our findings have clinical relevance, we examined the phosphorylation levels of GAC in NSCLC tissue array by immunohistochemistry using the anti-GAC-pS314 antibody. The tissue array includes lung cancer tissue samples and adjacent normal tissue from 75 NSCLC patients. The results demonstrated that 96% of tumor tissues expressed significantly higher levels of phosphorylated GAC than the adjacent normal tissues (Fig. 8a) and this was further validated by quantification of the staining (Fig. 8b). Representative staining of cancer and adjacent normal tissues are presented in Fig. 8c; expression levels of phosphorylated GAC (brown) were higher in cancer tissues with only weak or no expression observed in adjacent normal tissues. Statistical analysis of the quantified staining results from cancer tissues divided the cancer samples into two groups depending upon levels of GAC-pS314. Survival of the patients was then correlated to the GAC-pS314 levels. As shown in Fig. 8d, patients with low levels of phosphorylated GAC exhibited better survival than patients with high levels of phosphorylated GAC (*P* = 0.0207). These results indicate that the phosphorylation of GAC at Ser314 is closely related to NSCLC progression and patient survival.

## Discussion

Metabolic reprogramming in cancer cells has been intensively studied over the past decades. Aerobic glycolysis or the Warburg effect, a critical aspect of this altered metabolism, has been demonstrated to provide energy and intermediates for macromolecular biosynthesis to support rapid cell growth in cancer initiation and progression.^28,29^ Another alteration in metabolism is elevated utilization of glutamine. As with aerobic glycolysis, glutamine metabolism can also be used to satisfy bioenergetic and biosynthetic requirements for cancer cell growth.^4,5^ As an important metabolic enzyme in glutamine metabolism, glutaminase has received great interest.^3,30^ Glutaminase C, a splice variant of *GLS1*, has been demonstrated to be the major form of glutaminase in certain types of cancer cells.^8–11^ In B lymphoma and prostate cancer cells, the GLS1 expression level was upregulated by c-Myc though suppression of miR-23a/b.^16^ In our previous study, we found a novel signaling pathway in which GAC activity, but not expression levels could be regulated by Rho GTPases through NF-κB in transformed/cancer cells.^12^ Subsequently, Moeez et al. (2012) reported that the NF-κB p65 subunit could bind to the miR-23a promoter and inhibit miR-23a expression in leukemic cells, resulting in higher GLS1 expression.^31^ In breast cancer cells, ErbB2-mediated upregulation of KGA expression did not correlate to c-Myc expression, but correlated to the activation of the NF-κB pathway.^32^ Together, these studies provide strong evidence that NF-κB is another important regulator of glutaminase in addition to c-Myc. However, the precise molecular mechanism of NF-κB in regulating glutaminase activity remains unclear.

In this study, we demonstrated that downregulation of GAC expression by specific siRNAs or inhibition of GAC activity by the specific inhibitor 968 significantly reduced the growth of NSCLC cells but not HBE cells. These results are consistent with previous findings that glutamine-dependent NSCLC cells are more sensitive to GLS1 inhibition.^33^ We showed that the expression level of GAC varied between different NSCLC cells, some cancer cells showed higher expression of GAC while some showed even lower expression when compared with bronchial epithelia cells.^33,34^ However, we demonstrated that the GAC activity in NSCLC cells was much higher than that in HBE cells, indicating the dependence of NSCLC cells on glutamine. We also observed that phosphorylation is the major reason for the much higher GAC activity in NSCLC cells than that in HBE cells. We have identified Ser314 as the key phosphorylation site in GAC and found that this site is very important for GAC activity. Thangavelu et al. reported that the residue Ser314 was located in the glutaminase domain and this residue together with others formed a loop region (Glu312-Pro329) in the crystal structure of KGA. This loop is located near the active site. In the ligand-free structure, the loop region forms the closed conformation of the active site. Specifically, Phe318 makes hydrophobic interactions with Tyr466 and the side chain of the Asn319 makes hydrogen-bonding contact with the backbone of Asn335. The residues Tyr466 and Asn335 in the active site are involved in binding to L-glutamine and in catalysis.^14^ According to Ambrosio’s group, the structure-based activation mechanism is shared between KGA and GAC isoforms since they have the same glutaminase domain.^35^ Thus, we propose that phosphorylation of GAC on Ser314 might lead to elevated negative charge at the Glu312 to Pro329 loop and subsequent increase in the flexibility of the active site, allowing L-glutamine to enter into the active site to be catalyzed. When GAC is dephosphorylated, the structure might return back to a closed conformation.

Furthermore, we found that the phosphorylation of GAC could be directly regulated by PKCε. As a serine–threonine kinase, overexpression of PKCε has been demonstrated to be a hallmark of multiple cancers, including breast cancer, prostate cancer, and lung cancer.^36–39^ In NSCLC cells, PKCε is upregulated and knockdown of PKCε impairs tumor growth.^39^ However, the molecular mechanism by which PKCε regulates cancer metabolism has not been defined until our current work. We have shown that PKCε can affect the proliferation of NSCLC cells by regulating glutamine metabolism. We have shown that NF-κB binds to the promoter region and regulates the transcription of *PRKCE* gene such that PKCε in turn regulates glutaminase activity. Thus, we have demonstrated for the first time that the NF-κB-PKCε axis regulates glutaminase activity by modulating the phosphorylation level of GAC at Ser314 in NSCLC cells. Taken together, our findings reveal that the NF-κB-PKCε axis promotes tumorigenesis by upregulating glutamine metabolism and thus facilitating metabolic reprogramming to satisfy the bioenergetic and biosynthetic requirements of rapid cancer cell growth and metastasis.

Another important finding of our study is that blocking glutamine metabolism leads to tumor cell differentiation. We describe two types of tumors with differences in tissue structure and differential expression of genes representing tumor differentiation states. The high expression of Ki67 indicated that tumors derived from a parental A549 cell line proliferated rapidly. In contrast, tumors formed by A549-expressing mutant GAC (S314A) were Ki67-negative indicative of a very low proliferation rate. Previous studies demonstrated that absence of TTF-1 was pathognomonic of high-grade, poorly differentiated tumors. In addition, gain- and loss-of-function experiments in cells derived from metastatic and non-metastatic tumors demonstrate that TTF-1 controls tumor differentiation and metastatic potential in vivo.^26^ Another study showed that patients with adenocarcinomas with low or high TTF-1 expression had a significantly better outcome than those in which TTF-1 expression was absent.^40^ Tumors derived from parental A549 cells were negative for TTF-1 expression. On the contrary, TTF-1 expression was observed in tumors formed by A549-GAC (S314A) cells although the expression level was low. These findings suggest that inhibition of glutamine metabolism leads lung adenocarcinomas to differentiate from high-grade poorly differentiated tumors to well-differentiated tumors. To our knowledge, this is the first study linking glutamine metabolism to the differentiation states of lung adenocarcinoma.

To further study the mechanisms leading to different phenotypic outcomes in A549-GAC (S314A) cells, we performed metabolomic and human genome oligo microarray analyses. Our metabolomic studies revealed reduced production of glutamate and enhanced accumulation of glutamine, indicating impaired glutamine metabolism in A549-GAC (S314A) cells. This accords with the pathway analysis from a human genome oligo microarray that indicated the NF-κB signaling pathway to be significantly downregulated in A549-GAC (S314A) cells, clarifying the importance of NF-κB in glutamine metabolism. In addition, the production of glycolysis (lactate) and metabolites of the TCA cycle (succinate, fumarate) were reduced in A549-GAC (S314A) cells. This accords with the pathway analysis indicating that the glycolysis and carbon metabolism pathways were downregulated in A549-GAC (S314A) cells. The pathway analysis also showed that the MAPK pathway was downregulated. Correlated with this was the accumulation of precursors for nucleotide synthesis (UMP, IMP) in A549-GAC(S314A) cells, suggesting that cell cycle progression was impaired and the proliferation rate was reduced. Taken together, these metabolomic and human genome oligo microarray analyses demonstrated that blocking glutamine metabolism led to genetic reprogramming and subsequent metabolic reprogramming with the consequence of reduced proliferation rate and tumorigenesis of NSCLC cells.

Recently, it was reported that KGA activity was stimulated by EGF and regulated by Raf-1/Mek/Erk signaling in 293T cells.^14^ In NSCLC cells, GAC has been determined to be the predominant GLS1 isoform.^33^ Therefore, it is of important clinical relevance to elucidate the mechanism regulating GAC activity in NSCLC cells. Taking our results together, we conclude that GAC activity is enhanced in NSCLC cells and regulated by phosphorylation. The phosphorylated GAC is significantly upregulated in both NSCLC cell lines and patient tissues. Phosphorylation levels are closely related to the differentiation states of lung adenocarcinoma and the survival of NSCLC patients. Thus, these findings raise exciting possibilities regarding the targeting of glutaminase activity as a potential therapeutic strategy against non-small cell lung cancer.

## Materials and methods

### Cell culture

Human bronchial epithelial (HBE) cells were cultured in Airway epithelial cell basal medium using the bronchial/tracheal epithelial cell growth kit (ATCC). Human non-small cell lung cancer (NSCLC) cell lines (H23, H1299, A549, H292, and SPC-A1) and breast cancer cell lines (MDA-MB-231 and MCF7) were cultured in RPMI 1640 (Gibco) supplemented with 10% FBS (Gibco). Hepatocarcinoma cell lines (HepG2 and HCC-LM3) and the cervical cancer cell line (Hela) were cultured in DMEM (Gibco) supplemented with 10% FBS (Gibco). A549 cells in which the endogenous GAC carried the mutation S314A were produced by CRISPR/Cas9 mutagenesis by Beijing Biocytogen Co. Ltd. The cells were cultured in RPMI 1640 (Gibco) supplemented with 10% FBS (Gibco).

### Cell growth assay

To carry out cell growth assays under glutamine (Gln) free condition, cells were seeded in 12-well plates at a density of 10^5^ cells per well in 2 ml of complete culture medium. The medium was changed to Gln free medium supplemented with 10% FBS on the following day. Medium was changed every 2 days. After 6 days, the cell number was counted. For cell growth assays with 968 treatment, cells were seeded in 12-well plates at a density of 10^5^ cells per well in 2 ml medium with 10% FBS. On the following day, the medium was changed to RPMI 1640 + 10% FBS + DMSO, or RPMI 1640 + 10% FBS + 968. Medium containing 968 was changed every 2 days. After 6 days, the cell number was counted. For cell growth assays following knockdown of GAC or with overexpression of wild-type or mutant (S314A) GAC, cells were transiently transfected with GAC siRNAs or related plasmids and seeded in 24-well plates at 3000 cells per well in 0.5 ml medium containing 10% FBS. The medium was changed every 2 days. Cells were fixed in 3.7% formaldehyde at the indicated times and stained with 0.1% crystal violet. Dye was extracted with 10% acetic acid and the relative proliferation rate was assessed from the increase in absorbance at 595 nm.

For soft agar assays, 10^4^ cells were suspended in RPMI 1640 supplemented with 10% FBS and 0.3% agarose followed by plating on a solidified layer of RPMI 1640 supplemented with 0.5% agarose and 10% FBS. Fresh medium with 10% FBS and 0.5% agarose were added to the cells every week. Two weeks later, colonies larger than 50 μM were scored.

### Mitochondrial isolation and glutaminase activity assay

The detailed procedures for isolation of mitochondria and assaying glutaminase activity have been previously described.^12^ Briefly, mitochondrial were isolated using the mitochondria isolation kit from QIAGEN following the manufacturer’s instructions. Total of 2 × 10^7^ cells were collected and centrifuged at 500 × *g* for 10 min at 4 ℃. The cell pellets were suspended in 2 ml of lysis buffer and incubated on ice for 10 min using an end-over-end shaker. The cell lysates were centrifuged at 1000 × *g* for 10 min at 4 ℃ and the resulting pellets were resuspended in 1.5 ml disruption buffer using a blunt-ended, 23-gauge needle, and a syringe. The suspension was centrifuged at 6000 × *g* for 20 min at 4 ℃. The pellets were resuspended in 100 μl of storage buffer and assayed for glutaminase activity. Briefly, 20 μl resuspended mitochondrial lysate was added to reaction buffer I [57 mM Tris-acetate (pH 8.6), 0.225 mM EDTA, 17 mM glutamine] and incubated at 37 ℃ for 1 h while rotating. The reaction was stopped by adding 10 μl ice-cold 3 M hydrogen chloride (HCl) and incubated on ice for 5 min. Then, 10 μl of the quenched reaction mixture was added to a reaction buffer II containing 114 mM Tris-HCl (pH 9.4), 0.35 mM adenosine diphosphate (ADP), 1.7 mM nicotinamide adenine dinucleotide (NAD), 6.3 U/ml glutamate dehydrogenase, 1% hydrazine to give a final volume of 230 μl, and incubated at room temperature for 45 min. The formation of NADH was detected by absorbance at 340 nm. Measurements were carried out in triplicate. For the glutaminase activity assay upon GAC-WT and GAC mutants, the indicated plasmids were transiently transfected into H1299 cells and the tagged proteins were immunoprecipitated from cell extracts using an anti-V5 antibody. Assay procedures were as above.

### RNA interference

The detailed procedures for RNAi were carried out as previously described.^12^ The GAC siRNAs we used were:

siRNA1 forward sequence: 5′-UAAUUGGGCAGAAACCACCAUUAGC-3′,

siRNA1 reverse sequence: 5′-GCUAAUGGUGGUUUCUGCCCAAUUA-3′;

siRNA2 forward sequence: 5′-UUAACAGCAAUUGCAUAUUUCAGGG-3′,

siRNA2 reverse sequence: 5′-CCCUGAAAUAUGCAAUUGCUGUUAA-3′.

### Immunoprecipitation

The detailed procedures for immunoprecipitation have been previously described.^41^

### Luciferase activity assay

Human genomic DNA was extracted from H1299 cells and the PKCε promoter fragment (1163 bp) was amplified by PCR and cloned into the KpnI/XhoI restriction sites of pGL3-enhancer vector. The pGL3-enhancer vector containing PKCε promoter fragment with mutations in NF-κB binding site was constructed by Bio-Transduction Lab Co., Ltd. For transient transfections, the pGL3-enhancer vector containing the PKCε promoter fragment was transfected into H1299 and A549 cells using SuperFectin II in vitro DNA transfection reagent (Shanghai Pufei Biotech). Forty-eight hours after transfection, cells were lysed and luciferase activity was detected using the Dual-Luciferase reporter assay kit (Promega). The relative levels of luciferase activity were normalized to the levels of luciferase activity of the Renilla control plasmid.

### Site-directed mutagenesis

QuikChange II XL site-directed mutagenesis kit (Agilent Technologies) was used for mutagenesis. pCDNA3.1-V5-GAC was used as a template to generate mutations of glutaminase phosphorylation sites. The following primers were used:

T112A: 5′-gccccggggaggcggacgcgttt-3′ (sense)

5′-aaacgcgtccgcctccccggggc-3′ (antisense)

T188A: 5′-atatgttaagattaactcttcaagcaacatcagatggtgtcatgcta-3′ (sense)

5′-tagcatgacaccatctgatgttgcttgaagagttaatcttaacatat-3′ (antisense)

S274A: 5′-gtagatggacagaggcatgctactggagataccaaag-3′ (sense)

5′-ctttggtatctccagtagcatgcctctgtccatctac-3′ (antisense)

T278A: 5′-gaggcattctactggagatgccaaagttcccttct-3′ (sense)

5′-agaagggaactttggcatctccagtagaatgcctc-3′ (antisense)

S314A: 5′-cgatatgttggaaaagagccggctggactaagattcaacaaact-3′ (sense)

5′-agtttgttgaatcttagtccagccggctcttttccaacatatcg-3′ (antisense)

S314D: 5′-cgatatgttggaaaagagccgaatggactaagattcaacaaact-3′ (sense)

5′-agtttgttgaatcttagtccattcggctcttttccaacatatcg-3′ (antisense)

S511A: 5′-cctctggataagatgggcaacgctgttaagggaattcacttttg-3′ (sense)

5′-caaaagtgaattcccttaacagcgttgcccatcttatccagagg-3′ (antisense)

S576A: 5′-gacagtatggaaaaaagtggcacctgagtcaaatgagga-3′ (sense)

5′-tcctcatttgactcaggtgccacttttttccatactgtc-3′ (antisense).

The correct resulting mutations were confirmed in the plasmids by DNA sequencing. PKCε (K437R) was used as the dominant-negative mutant as previously described.^42,43^ The pCMV-HA-PKCε (K437R) was made by Generay Biotech in Shanghai.

### Quantitative RT-PCR

Total RNA was extracted using TRIzol reagent (Invitrogen) and 1 μg total RNA was used for reverse transcription using the PrimeScript RT reagent kit with gDNA eraser (Takara) according to the manufacturer’s instructions. Quantitative RT-PCR was performed with SYBR Green dye. The relative amount of cDNA was calculated by the comparative Ct method using GAPDH as a control. The sequences of the probes used to quantify GAC mRNA levels were: 5′-AGGTGGTGATCAAAGGCATTC-3′ (sense); 5′-GCTTTTCTCTCCCAGACTTTCC-3′ (antisense). PCR reactions were performed in triplicate.

### Chromatin immunoprecipitation assay (ChIP)

Cells were fixed with 1% formaldehyde for 15 min at room temperature to cross-link DNA with associated proteins. Then, the buffer was adjusted to 0.125 M glycine to stop the cross-linking reaction. Cells were collected and lysed in SDS lysis buffer (50 mM Tris-HCl pH 8.0, 10 mM EDTA, 1% SDS, 1 mM PMSF) at 4 ℃ and sonicated to disrupt DNA (200–1000 bp). The sonicated DNA fragments were then diluted in buffer comprising 0.01% SDS, 1.1% Triton × 100, 1.2 mM EDTA, 16.7 mM Tris-HCl pH 8.0, 167 mM NaCl, and 1 mM PMSF and incubated with protein G-agarose beads for 8 h at 4 ℃ to pre-clean the sample. Equal amounts of the sample were then added to solutions containing anti-p65 or anti-acetyl histone H3 antibodies or control rabbit IgG and protein G-agarose beads and incubated overnight at 4 ℃. Ten percent of the sample was kept as input. The beads were washed with buffer comprising 50 mM Tris-HCl pH 7.5, 1 M NaCl, 1 mM EDTA, 1% NP-40, 1% Na-Deoxycholte, 0.1% SDS, 2 M urea, 1 mM PMSF, and 10 mM Tris-HCl pH 7.5. The beads were eluted with elution buffer containing 50 mM NaHCO_3_ and 1% SDS. Cross-linking was reversed by adjusting to 0.2 M NaCl and incubating overnight at 65 ℃. Then, a buffer containing 10 mM EDTA, 40 mM Tris-HCl, 20 μg proteinase K, 20 μg RNase A was added and incubated at 45 ℃ for 1 h and DNA was then extracted using a genomic DNA mini preparation kit with a spin column (Beyotime) and analyzed by PCR using the following primers:

5′-GAGCCGGATCGGCGG-3′ (sense)

5′-AAAATCCACAAGCCCCACG-3′ (antisense)

Primers specific for human GAPDH were used as control primers: 5′-TACTAGCGGTTTTACGGGCG-3′ (sense)

5′-TCGAACAGGAGGAGCAGAGAGCGA-3′ (antisense)

### Electrophoretic mobility shift assay (EMSA)

Nuclear extracts were prepared using a Nuclear and Cytoplasmic Extraction Kit (CWBIO). The LightShift Chemiluminescent EMSA Kit was purchased from Thermo Scientific (20148). The nuclear extracts were incubated with biotin-labeled DNA probes (20 fmol) or unlabeled probes (4 pmol) in 20 μl reaction buffer [1 × binding buffer, 2.5% glycerol, 5 mM MgCl_2_, 50 ng/μl Poly(dI-dC), 0.05% NP-40] for 20 min at room temperature before adding 5× loading buffer to the reaction mixture. The samples were subjected to 6.5% native polyacrylamide gel electrophoresis and transferred to a nylon membrane. The biotin end-labeled DNA was detected using the streptavidin-horseradish peroxidase conjugate and chemiluminescent substrate. Cold probe indicated the DNA probe without biotin label.

The sequence of the probe for the WT NF-κB binding site is (underlined):

Sense: 5′-CGAGCCCGCGCGGATGTGAGATTCCGGGCTCCTGGCGCCT-3′

Antisense: 5′-AGGCGCCAGGAGCCCGGAATCTCACATCCGCGCGGGCTCG-3′

The sequence of the probe for the mutant of the NF-κB binding site is (underlined):

Sense: 5′-CGAGCCCGCGCGGATGTTAGATGTTGGGCTCCTGGCGCCT-3′

Antisense: 5′-AGGCGCCAGGAGCCCAACATCTAACATCCGCGCGGGCTCG-3′

### Western blot

Protein extracts were prepared using NP-40 lysis buffer containing phosphatase and protease inhibitors and the cell lysates were then subjected to Laemmli SDS-PAGE followed by immunoblot using indicated antibodies. The anti-GAC antibody was purchased from Abcam (ab93434). The anti-phospho-serine 314 specific polyclonal antibody against GAC (GAC-pS314) was made by Shanghai Genomics Inc (antigen sequence: YVGKEPS(p)GLRFNK-C; immunogen: Peptide-KLH conjugated). Isoelectric focusing was conducted as previously described^44^ followed by immunoblot with the anti-V5 antibody.

### In vitro PKC kinase assay

The detailed procedures for the in vitro PKC kinase assay were as previously described with minor modification.^42^ Briefly, the indicated plasmids were separately transfected into HBE cells and the cells were lysed using PKC extraction buffer (50 mM HEPES, pH 7.5, 150 mM NaCl, 0.1% Tween 20, 1 mM EDTA, 2.5 mM EGTA, 10% glycerol) containing protease inhibitors (10 µg/ml aprotinin, 10 µg/ml leupeptin, 0.1 mM phenylmethylsulfonylfluoride) and phosphatase inhibitors (1 mM NaF, 0.1 mM Na_3_VO_4_, 10 mM β-glycerophosphate). HA-PKCε and HA-PKCε (K437R) were immunoprecipitated using anti-HA antibody and protein G-agarose. The V5-GAC and V5-GAC (S314A) were immunoprecipitated using an anti-V5 antibody and protein G-agarose. After a 4 h incubation at 4 ℃, the immunoprecipitates were washed twice with PKC extraction buffer followed by three washes with IP kinase buffer (50 mM HEPES, pH 7.5, 10 mM MgCl_2_, 1 mM DTT, 2.5 mM EGTA, 1 mM NaF, 0.1 mM Na_3_VO_4_, 10 mM β-glycerophosphate). The immunoprecipitates containing HA-PKCε or HA-PKCε (K437R) were resuspended in 20 μl IP kinase buffer and added to 40 μl IP kinase buffer containing V5-GAC or V5-GAC (S314A) and 1 mM ATP. The reactions were carried out at 30 ℃ for 30 min and then terminated by adding SDS sample buffer before boiling for 10 min. The phosphorylation states of GAC were examined using manganese (II)-Phos-tag^TM^ SDS-PAGE. The Phos-tag^TM^ was purchased from Wako Chemicals (AAL-107). The Phos-tag SDS-PAGE was performed on 8% (w/v) acrylamide gels containing 50 μM Phos-tag acrylamide and 100 μM MnCl_2_. The separated proteins were transferred to PVDF membranes and detected using anti-GAC antibody.

### In vivo xenograft assay

Cell suspensions (1 × 10^7^ cells) in a total volume of 100 μl were injected subcutaneously into the flanks of 3–4-week-old male BALB/C nude mice (SLAC, Shanghai). Four weeks after the injection, the mice were killed. Tumors were dissected out and their weights and volumes were measured. Tumor volume was calculated using the formula: volume (mm^3^) = π/6 × (large diameter) × (smaller diameter)^2^. All mice were housed in the SPF animal facility of the Institute of Translational Medicine at Nanchang University.

### GLS (S314A) mutation by CRISPR/Cas9 system

The GLS (S314A) mutant knockin A549 cell line was prepared by Beijing Biocytogen Co. Ltd. using EGETM system (Extreme Genome Editing System), which utilizes Cas9/sgRNA mutagenesis. The resulting cells were homozygous for the required mutation. Serine 314 of the GLS gene was replaced with alanine. Cas9/sgRNA plasmid was designed against exon 6 of the human *GLS* gene. The sgRNA sequence used is GTTGAATCTTAGTCCACT.

Primers for amplifying the 5′ homologous arm:

5′-TTTAAGAAGGAGATATACATGTTGGGACATTGTTCAGGGAACCTGG-3′

5′-AAACTCATCAATGTATCTTAACAATGATATAGCATTTGCATATAACC-3′

Primers for amplifying the 3′ homologous arm:

5′-GATATGTTGGAAAAGAGCCGGCTGGACTAAGATTCAACAAAC-3′

5′-TTGTTAGCAGCCGGATCTCAGTCACAGCCATTACTACAAACTTCGC-3′

A total of 2 × 10^6^ cells were transfected by electroporation (10 μg plasmid/1 × 10^6^ cells) and then seeded in 100 mm culture dishes. One day after transfection, puromycin (2 μg/ml) was added to the culture medium, to allow single cell-derived colony formation. After puromycin selection, surviving clones were picked and expanded for genotyping.

### Global cDNA microarray analysis

An Agilent whole human genome oligo microarray was used and analyzed in KangChen Bio-tech. Differentially expressed genes were identified through “fold-change” screening.

### Measurements of metabolites

A glutamine and glutamate determination kit was purchased from Sigma (GLN1). An ATP determination kit was purchased from Thermo Fisher (AZZ066). The lactic acid assay kit was purchased from the Nanjing Jiancheng Bioengineering Institute (A019-2). All assays were performed following protocols described in the respective instruction manuals. NMR analysis for the concentrations of different metabolites was conducted in Wuhan Anachro Technologies INC.

### Immunohistochemistry

Lung adenocarcinoma tissue microarray analysis was purchased from the National Engineering Center for BioChips in Shanghai, China. A more complete description of the human specimens is included in Supplementary Table S11. The expression of phosphorylated GAC in the tissue was evaluated by immunohistochemical staining with an anti-GAC-pS314 antibody. The tissue microarray slide was deparaffinized, rehydrated, and subjected to an epitope retrieval step. Subsequently, 6% hydrogen peroxide was used to block endogenous peroxidase activity. The slide was washed three times in PBS and then incubated with the anti-GAC-pS314 antibody at 4 ℃ overnight. After three washes in PBS, the slide was incubated with horseradish peroxidase-conjugated secondary antibody for 1 h. The stain was developed with either chromogen or haematoxylin solutions. The images of the tissue microarray were analyzed by ImageScope software. The immunohistochemical staining of tumors derived from the parental A549 and A549-GAC(S314A) cells was performed as indicated above. Anti-Ki67 and anti-TTF1 antibodies were purchased from Abcam (ab15580 and ab76013). Micrographs were obtained using an Olympus IX71 microscope.

### Statistical analysis

Data are presented as means ± SD. Statistical comparisons were made by ANOVA or a two-tailed Student’s *t* test; *P* values ≤0.05 was considered statistically significant. Differentially expressed genes were identified through “fold-change” screening and the threshold was a fold-change ≥2.

## Electronic supplementary material

Figure S1

Figure S2

Figure S3

Figure S4

Figure S5

Figure S6

Figure S7

Figure S8

Figure S9

Figure S10

Figure S11

Figure S12

Supplementary table 1

Supplementary table 2

Supplementary table 3

Supplementary table 4

Supplementary table 5

Supplementary table 6

Supplementary table 7

Supplementary table 8

Supplementary table 9

Supplementary table 10

Supplementary table 11

licence

## Electronic supplementary material

**Supplementary information** accompanies for this paper at 10.1038/s41422-018-0021-y.
