# Supplementary material for: Phosphorylation of glutaminase by PKCε is essential for its enzymatic activity and critically contributes to tumorigenesis
Source: Cell Res. 2018 Mar 7;28(6):655–69. doi: 10.1038/s41422-018-0021-y (PMC5993826; doi:10.1038/s41422-018-0021-y)
Supplement: Supplementary file 1 — Figure S1 [file 41422_2018_21_MOESM1_ESM.pdf]

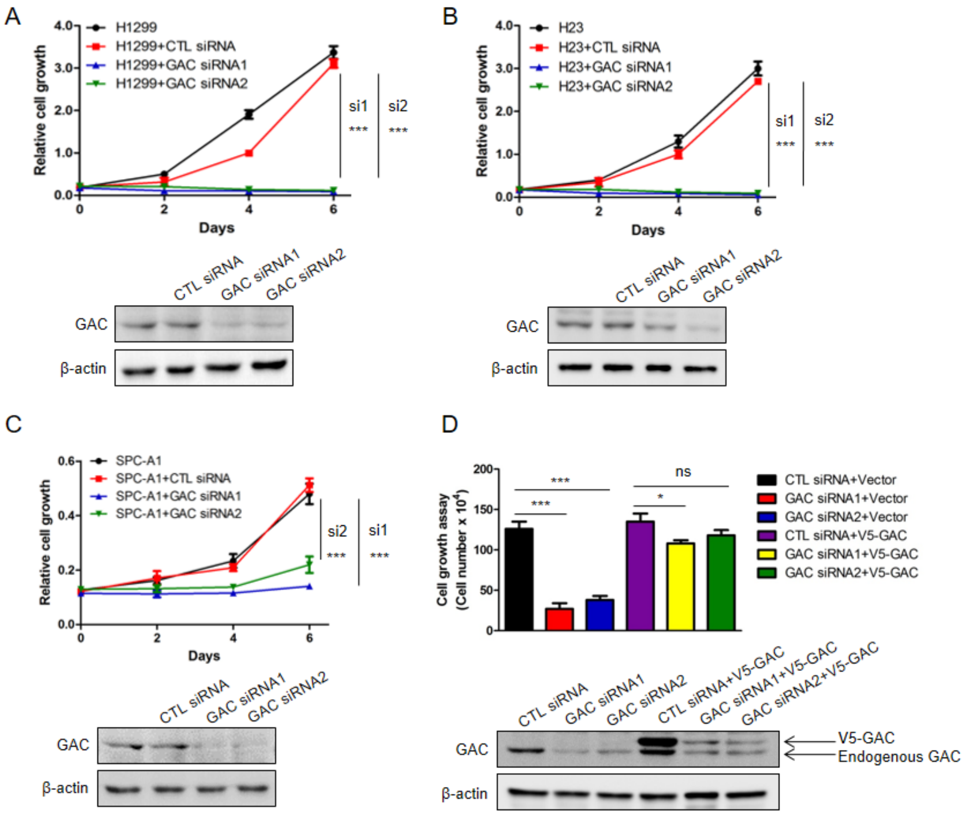
A
Relative cell growth
Days
H1299
H1299+CTL siRNA
H1299+GAC siRNA1
H1299+GAC siRNA2
si1
***
si2
***
CTL siRNA
GAC siRNA1
GAC siRNA2
GAC
β-actin
B
Relative cell growth
Days
H23
H23+CTL siRNA
H23+GAC siRNA1
H23+GAC siRNA2
si1
***
si2
***
CTL siRNA
GAC siRNA1
GAC siRNA2
GAC
β-actin
C
Relative cell growth
Days
SPC-A1
SPC-A1+CTL siRNA
SPC-A1+GAC siRNA1
SPC-A1+GAC siRNA2
si2
***
si1
***
CTL siRNA
GAC siRNA1
GAC siRNA2
GAC
β-actin
D
Cell growth assay
(Cell number x 10⁴)
***
***
ns
*
CTL siRNA+Vector
GAC siRNA1+Vector
GAC siRNA2+Vector
CTL siRNA+V5-GAC
GAC siRNA1+V5-GAC
GAC siRNA2+V5-GAC
CTL siRNA
GAC siRNA1
GAC siRNA2
CTL siRNA+V5-GAC
GAC siRNA1+V5-GAC
GAC siRNA2+V5-GAC
GAC
β-actin
V5-GAC
Endogenous GAC

Supplementary information, Figure S1. Glutaminase is important for the growth of NSCLC. (A-C) NSCLC cells (H1299, H23 and SPC-A1) were cultured in RPMI 1640 with 10% FBS. Cells were transiently transfected with either control siRNA or GAC siRNAs. At the indicated times, cells were fixed in 3.7% formaldehyde and stained with 0.1% crystal violet. Dye was extracted with 10% acetic acid and the relative proliferation was determined by the absorbance at 595nm. Data represent the average of three independent experiments (mean ±SD). $^{***}P$<0.001 (Top figures). The knockdown efficiency of the siRNAs targeting GAC was determined by western blotting using an anti-GAC antibody (Bottom figures). **(D)** H1299 cells were co-transfected with control siRNA (CTL siRNA) or GAC siRNAs and empty vector or pcDNA3.1-V5-GAC (V5-GAC). 24 hours later, cells were seeded in 12-well plates at 10^5 cells per well in 2 ml complete culture medium. Medium was changed every 2 days. After 6 days, cell number was counted. Data represent the average of three independent experiments (mean ±SD). $^{*}P$<0.05, $^{***}P$<0.001, ns: P>0.05 (Top figure). The protein expression was determined by western blotting using indicated antibodies (Bottom figure).
