# Supplementary material for: Phosphorylation of glutaminase by PKCε is essential for its enzymatic activity and critically contributes to tumorigenesis
Source: Cell Res. 2018 Mar 7;28(6):655–69. doi: 10.1038/s41422-018-0021-y (PMC5993826; doi:10.1038/s41422-018-0021-y)
Supplement: Supplementary file 2 — Figure S2 [file 41422_2018_21_MOESM2_ESM.pdf]

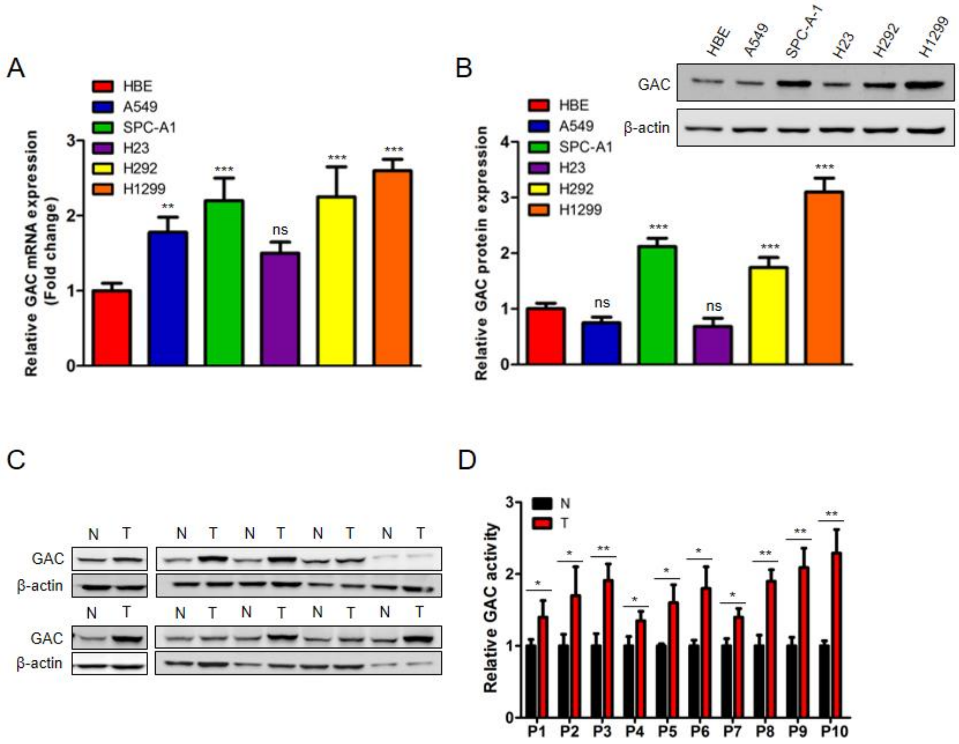

A
HBE
A549
SPC-A1
H23
H292
H1299
Relative GAC mRNA expression (Fold change)
**
***
ns
***
***
B
HBE
A549
SPC-A-1
H23
H292
H1299
GAC
β-actin
Relative GAC protein expression
ns
***
ns
***
***
C
N
T
GAC
β-actin
D
N
T
Relative GAC activity
P1
P2
P3
P4
P5
P6
P7
P8
P9
P10

Supplementary information, Figure S2. Glutaminase C activity is elevated in NSCLC. (A) The mRNA levels of GAC in NSCLC cell lines and HBE cells were determined by qPCR. Data represent the average of three independent experiments (mean ±SD). $^{**}P$<0.01, $^{***}P$<0.001, ns: *P*>0.05. **(B)** The expression of GAC in NSCLC cell lines and HBE cells was determined by western blotting with indicated antibodies (Top figure). Relative GAC expression over β-actin was quantified. Data represent the average of three independent experiments (mean ±SD). $^{***}P$<0.001, ns: *P*>0.05 (Bottom figure). **(C)** The expression of GAC in tumor tissues (T) and adjacent normal tissues (N) from 10 NSCLC patients was determined by western blotting with indicated antibodies. **(D)** Mitochondrial fractions were isolated from tumor tissues (T) and adjacent normal tissues (N) from 10 NSCLC patients, and glutaminase activity assay was performed. Data represent the average of three independent experiments (mean ±SD). $^{*}P$<0.05, $^{**}P$<0.01.
