# Supplementary material for: Phosphorylation of glutaminase by PKCε is essential for its enzymatic activity and critically contributes to tumorigenesis
Source: Cell Res. 2018 Mar 7;28(6):655–69. doi: 10.1038/s41422-018-0021-y (PMC5993826; doi:10.1038/s41422-018-0021-y)
Supplement: Supplementary file 3 — Figure S3 [file 41422_2018_21_MOESM3_ESM.pdf]

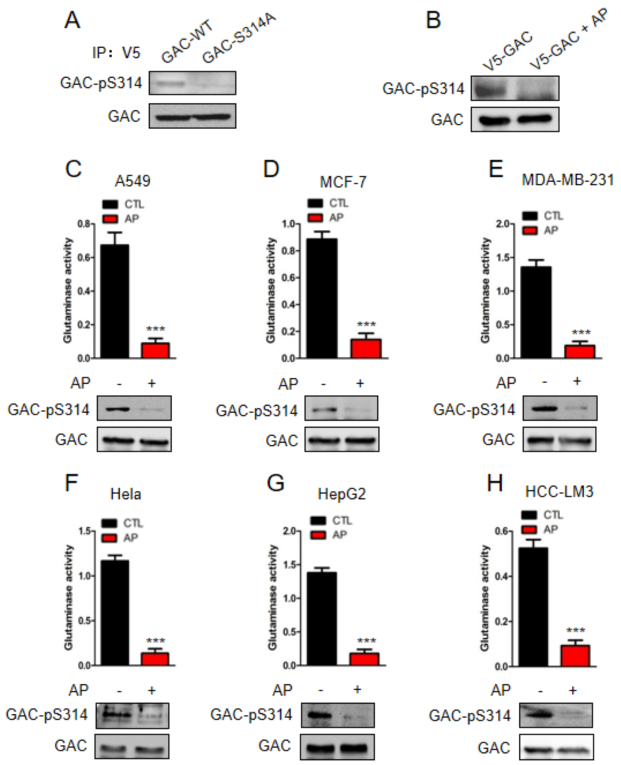
A
IP：V5
GAC-WT
GAC-S314A
GAC-pS314
GAC
B
V5-GAC
V5-GAC + AP
GAC-pS314
GAC
C
A549
D
MCF-7
E
MDA-MB-231
F
Hela
G
HepG2
H
HCC-LM3
CTL
AP
Glutaminase activity
***
AP - +
GAC-pS314
GAC

Supplementary information, Figure S3. The alkaline phosphatase treatment inhibits glutaminase activity in different types of cancer cells. (A) H1299 cells transiently transfected with V5-tagged GAC-WT or GAC-S314A were lysed and immunoprecipitated using an anti-V5 antibody. The lysates were blotted using indicated antibodies. **(B)** The PVDF membrane from Figure 2B was striped and detected by western blotting using an anti-GAC-pS314 antibody. **(C-H)** Lung cancer cells (A549), breast cancer cells (MDA-MB-231, MCF-7), cervical cancer cells (Hela) and hepatocarcinoma cells (HepG2, HCC-LM3) were lysed for immunoprecipitation using an anti-GAC antibody. The samples were treated with or without alkaline phosphatase (AP) for 1 hour at 37℃, then the glutaminase activity was assayed. Data represent the average of three independent experiments (mean±SD). *** $P<0.001$. Immunoblotting was done using indicated antibodies.
