# Supplementary material for: Phosphorylation of glutaminase by PKCε is essential for its enzymatic activity and critically contributes to tumorigenesis
Source: Cell Res. 2018 Mar 7;28(6):655–69. doi: 10.1038/s41422-018-0021-y (PMC5993826; doi:10.1038/s41422-018-0021-y)
Supplement: Supplementary file 4 — Figure S4 [file 41422_2018_21_MOESM4_ESM.pdf]

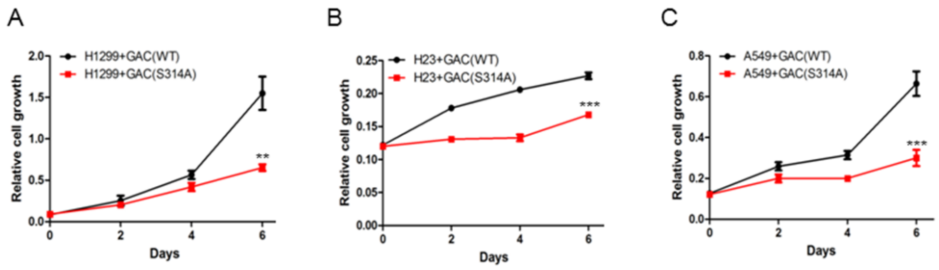
A
H1299+GAC(WT)
H1299+GAC(S314A)
Relative cell growth
Days
**
B
H23+GAC(WT)
H23+GAC(S314A)
Relative cell growth
Days
***
C
A549+GAC(WT)
A549+GAC(S314A)
Relative cell growth
Days
***

Supplementary information, Figure S4. GAC phosphorylation at S314 plays a key role in the growth of NSCLC cells. (A-C) H1299, H23 and A549 cells transiently transfected with GAC(WT) or GAC(S314A) mutant were cultured in RPMI 1640 with 10% FBS for indicated times, and then cells were fixed in 3.7% formaldehyde and stained with 0.1% crystal violet. Dye was extracted with 10% acetic acid and the relative proliferation was assessed from the increase in absorbance at 595nm. Data represent the average of three independent experiments (mean ±SD). $^{**}P<0.01$, $^{***}P<0.001$.
