# Supplementary material for: Phosphorylation of glutaminase by PKCε is essential for its enzymatic activity and critically contributes to tumorigenesis
Source: Cell Res. 2018 Mar 7;28(6):655–69. doi: 10.1038/s41422-018-0021-y (PMC5993826; doi:10.1038/s41422-018-0021-y)
Supplement: Supplementary file 5 — Figure S5 [file 41422_2018_21_MOESM5_ESM.pdf]

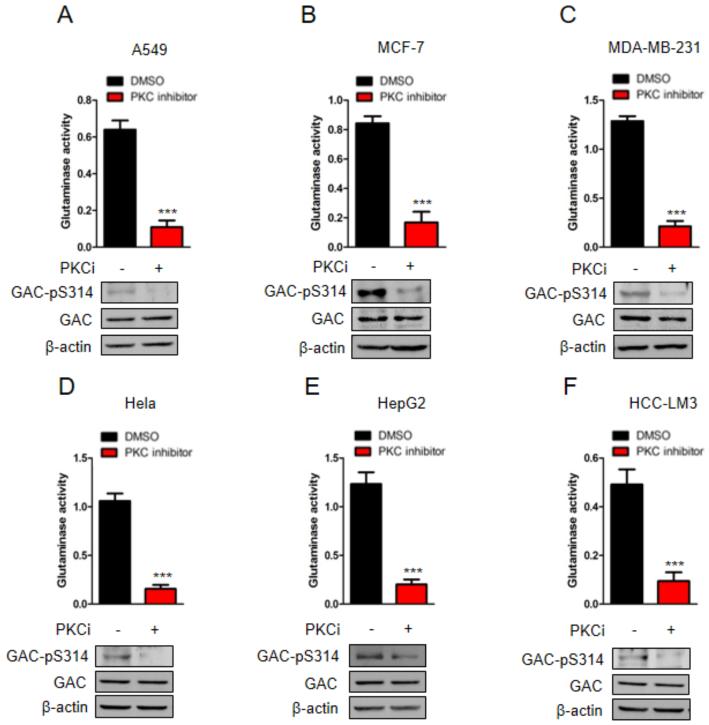
A
A549
DMSO
PKC inhibitor
Glutaminase activity
***
PKCi
-
+
GAC-pS314
GAC
β-actin
B
MCF-7
C
MDA-MB-231
D
Hela
E
HepG2
F
HCC-LM3

Supplementary information, Figure S5. PKCε inhibitor blocks glutaminase activity in different types of cancer cells. (A-F) Lung cancer cells (A549), breast cancer cells (MDA-MB-231, MCF-7), cervical cancer cells (Hela) and hepatocarcinoma cells (HepG2, HCC-LM3) were treated with PKCε inhibitor (Staurosporine, 50nM) for 24 hours. Then, mitochondria were isolated and glutaminase activity was examined. Data represent the average of three independent experiments (mean ±SD). $^{***}P<0.001$. The expression of indicated proteins was examined by western blotting.
