# Supplementary material for: Phosphorylation of glutaminase by PKCε is essential for its enzymatic activity and critically contributes to tumorigenesis
Source: Cell Res. 2018 Mar 7;28(6):655–69. doi: 10.1038/s41422-018-0021-y (PMC5993826; doi:10.1038/s41422-018-0021-y)
Supplement: Supplementary file 6 — Figure S6 [file 41422_2018_21_MOESM6_ESM.pdf]

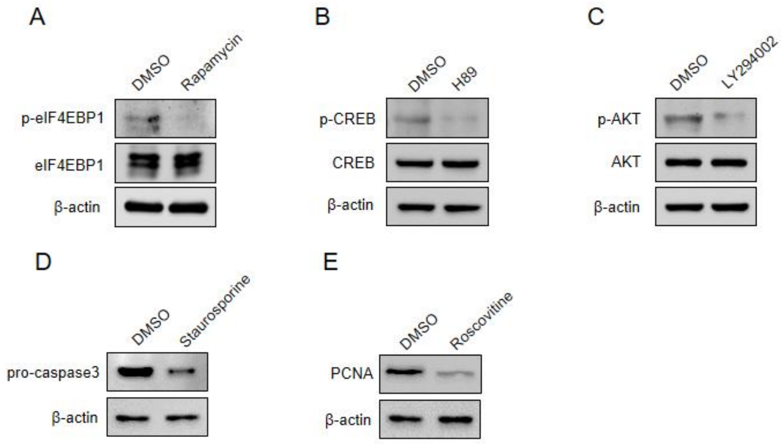
A
DMSO
Rapamycin
p-eIF4EBP1
eIF4EBP1
β-actin
B
DMSO
H89
p-CREB
CREB
β-actin
C
DMSO
LY294002
p-AKT
AKT
β-actin
D
DMSO
Staurosporine
pro-caspase3
β-actin
E
DMSO
Roscovitine
PCNA
β-actin

Supplementary information, Figure S6. Different kinase inhibitors work well in H1299 cells. (A-E) H1299 cells were treated with mTOR inhibitor (Rapamycin, 100nM), PKA inhibitor (H89, 20μM), AKT inhibitor (LY294002, 10μM), general PKC inhibitor (Staurosporine, 50nM) and CDK1 inhibitor (Roscovitine, 15μM) for 24 hours. Then, the cells were lysed and the expressions of indicated proteins were determined by western blotting.
