# Supplementary material for: Phosphorylation of glutaminase by PKCε is essential for its enzymatic activity and critically contributes to tumorigenesis
Source: Cell Res. 2018 Mar 7;28(6):655–69. doi: 10.1038/s41422-018-0021-y (PMC5993826; doi:10.1038/s41422-018-0021-y)
Supplement: Supplementary file 7 — Figure S7 [file 41422_2018_21_MOESM7_ESM.pdf]

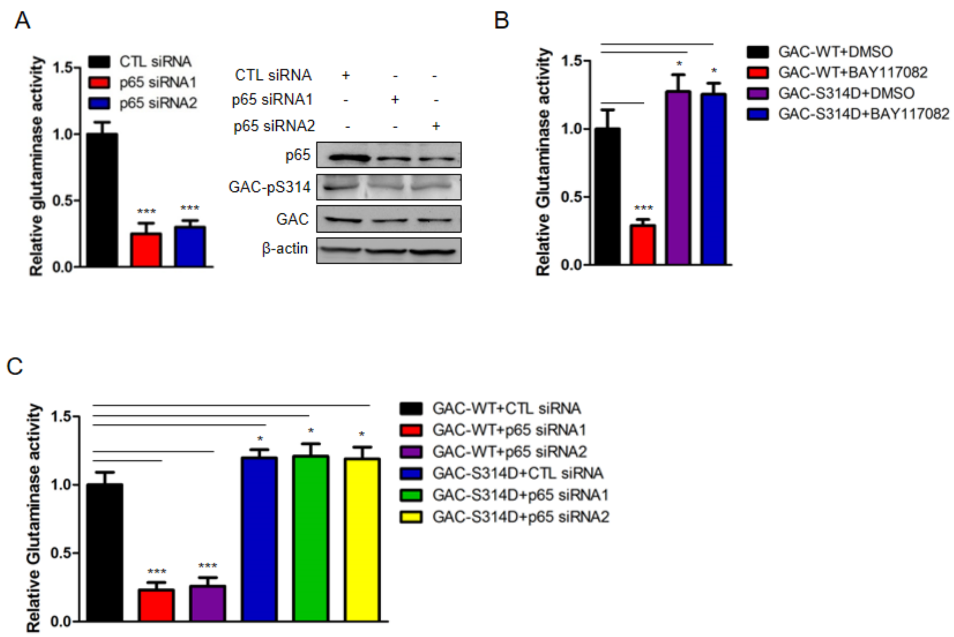
A
Relative glutaminase activity
CTL siRNA
p65 siRNA1
p65 siRNA2
***
***
CTL siRNA + - -
p65 siRNA1 - + -
p65 siRNA2 - - +
p65
GAC-pS314
GAC
β-actin
B
Relative Glutaminase activity
GAC-WT+DMSO
GAC-WT+BAY117082
GAC-S314D+DMSO
GAC-S314D+BAY117082
***
*
*
C
Relative Glutaminase activity
GAC-WT+CTL siRNA
GAC-WT+p65 siRNA1
GAC-WT+p65 siRNA2
GAC-S314D+CTL siRNA
GAC-S314D+p65 siRNA1
GAC-S314D+p65 siRNA2
***
***
*
*
*
0.0
0.5
1.0
1.5

Supplementary information, Figure S7. NF-κB regulates the phosphorylation of GAC at Ser314. (A) A549 cells were transiently transfected with control siRNA (CTL siRNA) or NF-κB (p65) siRNAs and mitochondria were isolated. Then the glutaminase activity was detected. Data represent the average of three independent experiments (mean ±SD). $^{***}P<0.001$ (Left figure). Cells transfected with control or p65 siRNAs were lysed and probed using indicated antibodies (Right figure). **(B)** H1299 cells were transiently transfected with V5-tagged GAC-WT or GAC-S314D. 24 hours later, the cells were treated with or without 5μM Bay117082 for 24 hours and lysed for immunoprecipation using the anti-V5 antibody. The glutaminase activity assay was then performed. Data represent the average of three independent experiments (mean ±SD). $^{*}P<0.05$, $^{***}P<0.001$. **(C)** H1299 cells were co-transfected with indicated siRNAs and GAC-WT or GAC-S314D and then lysed for immunoprecipation using the anti-V5 antibody, and the glutaminase activity assay was done. Data represent the average of three independent experiments (mean ±SD). $^{*}P<0.05$, $^{***}P<0.001$.
