# Supplementary material for: Phosphorylation of glutaminase by PKCε is essential for its enzymatic activity and critically contributes to tumorigenesis
Source: Cell Res. 2018 Mar 7;28(6):655–69. doi: 10.1038/s41422-018-0021-y (PMC5993826; doi:10.1038/s41422-018-0021-y)
Supplement: Supplementary file 8 — Figure S8 [file 41422_2018_21_MOESM8_ESM.pdf]

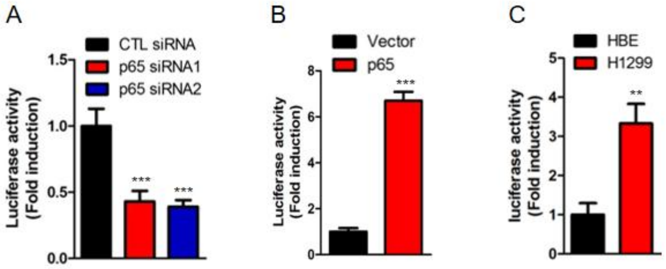

A
CTL siRNA
p65 siRNA1
p65 siRNA2
Luciferase activity
(Fold induction)
1.5
1.0
0.5
0.0
***
***
B
Vector
p65
Luciferase activity
(Fold induction)
8
6
4
2
0
***
C
HBE
H1299
luciferase activity
(Fold induction)
5
4
3
2
1
0
**

Supplementary information, Figure S8. PKCε is a new downstream target of NF-κB. (A) A549 cells were co-transfected with pGL3-enhancer vector containing PKCε promoter, Renilla control plasmid, and control siRNA (CTL siRNA) or NF-κB (p65) siRNAs. The relative levels of luciferase activity were normalized to the levels in untreated cells and to the levels of luciferase activity of the Renilla control plasmid. Data represent the average of three independent experiments (mean ±SD). $^{***}P<0.001$. **(B)** A549 cells were co-transfected with pGL3-enhancer vector containing PKCε promoter fragment, Renilla control plasmid and pcDNA3.0 empty vector or pcDNA3.0-p65 plasmid. The relative levels of luciferase activity were normalized to the levels in cells transfected with vector control and to the levels of luciferase activity of the Renilla control plasmid. Data represent the average of three independent experiments (mean ±SD). $^{***}P<0.001$. **(C)** pGL3-enhancer vector containing PKCε promoter fragment was transfected into HBE or H1299 cells. The relative levels of luciferase activity were normalized to the levels in HBE cells and to the levels of luciferase activity of the Renilla control plasmid. Data represent the average of three independent experiments (mean ±SD). $^{**}P<0.01$.
