# Supplementary material for: Phosphorylation of glutaminase by PKCε is essential for its enzymatic activity and critically contributes to tumorigenesis
Source: Cell Res. 2018 Mar 7;28(6):655–69. doi: 10.1038/s41422-018-0021-y (PMC5993826; doi:10.1038/s41422-018-0021-y)
Supplement: Supplementary file 9 — Figure S9 [file 41422_2018_21_MOESM9_ESM.pdf]

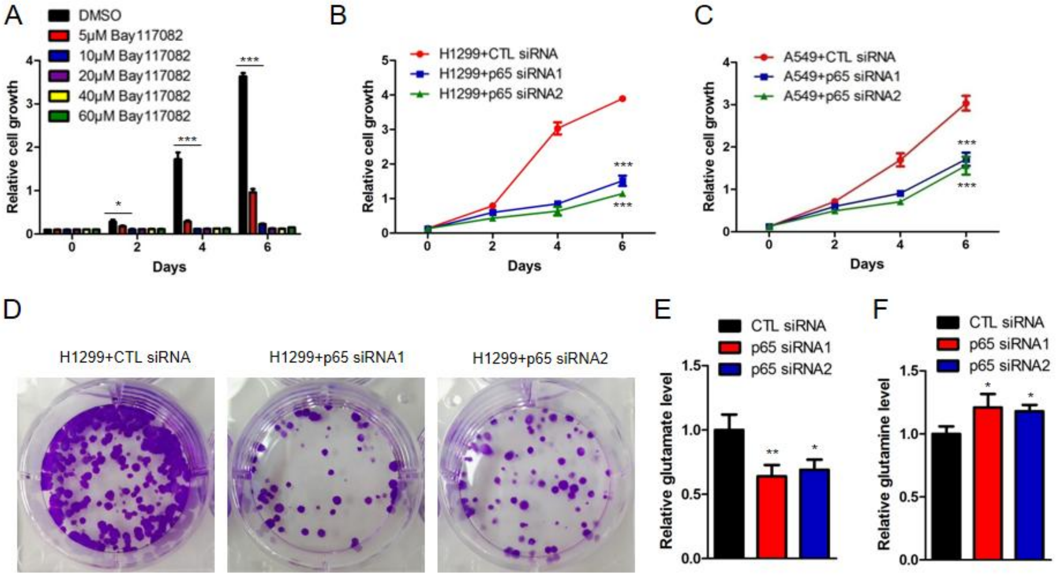
A
DMSO
5μM Bay117082
10μM Bay117082
20μM Bay117082
40μM Bay117082
60μM Bay117082
Relative cell growth
Days
B
H1299+CTL siRNA
H1299+p65 siRNA1
H1299+p65 siRNA2
Relative cell growth
Days
C
A549+CTL siRNA
A549+p65 siRNA1
A549+p65 siRNA2
Relative cell growth
Days
D
H1299+CTL siRNA
H1299+p65 siRNA1
H1299+p65 siRNA2
E
CTL siRNA
p65 siRNA1
p65 siRNA2
Relative glutamate level
F
CTL siRNA
p65 siRNA1
p65 siRNA2
Relative glutamine level

Supplementary information, Figure S9. Blocking p65 inhibits the growth and glutamine metabolism of NSCLC cells. (A) H1299 cells were treated with different concentrations of Bay117082 for indicated times, and then cells were fixed in 3.7% formaldehyde and stained with 0.1% crystal violet. Dye was extracted with 10% acetic acid and the relative proliferation was determined by the absorbance at 595nm. Data represent the average of three independent experiments (mean±SD). $^{*}P<0.05$, $^{***}P<0.001$. **(B and C)** H1299 (B) and A549 (C) cells were transiently transfected with either control siRNA (CTL siRNA) or p65 siRNAs. 24 hours later, the cells were seeded in 24-well plates at 3000 cells per well in 0.5 ml medium with 10% FBS. At the indicated times, cells were fixed in 3.7% formaldehyde and stained with 0.1% crystal violet. Dye was extracted with 10% acetic acid and the relative proliferation was determined by the absorbance at 595nm. Data represent the average of three independent experiments (mean±SD). $^{***}P<0.001$. **(D)** H1299 cells were transiently transfected with either control siRNA (CTL siRNA) or p65 siRNAs. 24 hours later, the cells were seeded in 6-well plate at 500 cells per well in 2 ml medium with 10% FBS. 10 days later, cells were fixed in 3.7% formaldehyde and stained with 0.1% crystal violet. **(E and F)** H1299 cells were transiently transfected with either control siRNA (CTL siRNA) or p65 siRNAs. 48 hours later, the cells were collected and glutamate (E) and glutamine (F) levels were determined by different kits. Data represent the average of three independent experiments (mean±SD). $^{*}P<0.05$, $^{**}P<0.01$.
