# Supplementary material for: Phosphorylation of glutaminase by PKCε is essential for its enzymatic activity and critically contributes to tumorigenesis
Source: Cell Res. 2018 Mar 7;28(6):655–69. doi: 10.1038/s41422-018-0021-y (PMC5993826; doi:10.1038/s41422-018-0021-y)
Supplement: Supplementary file 10 — Figure S10 [file 41422_2018_21_MOESM10_ESM.pdf]

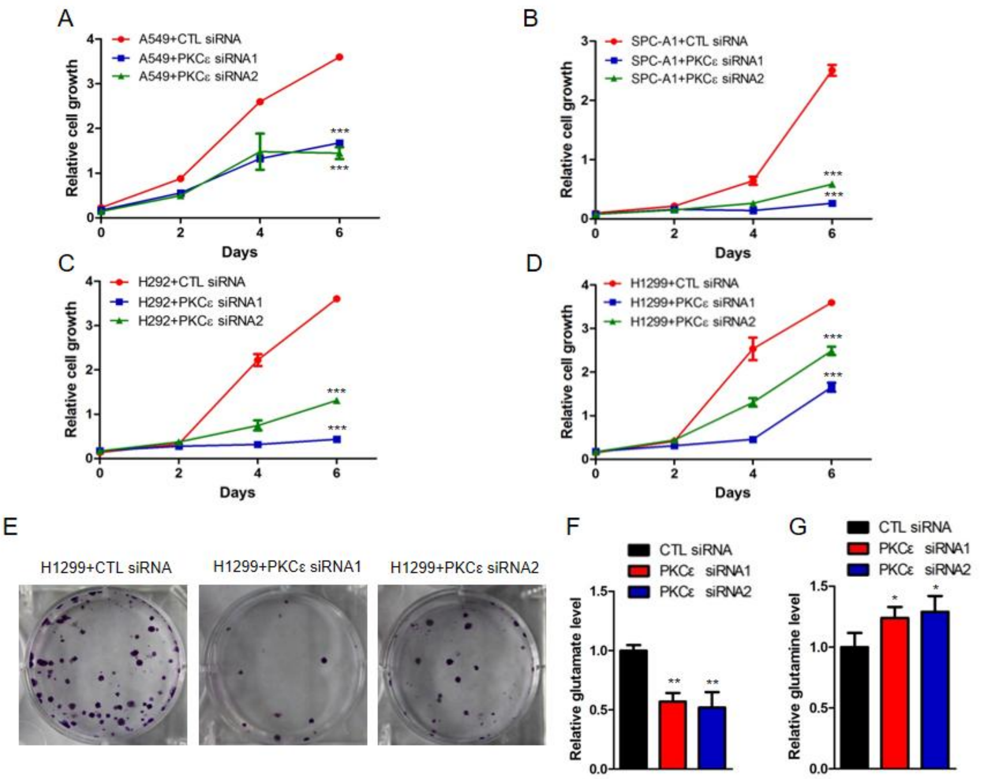

A
A549+CTL siRNA
A549+PKCε siRNA1
A549+PKCε siRNA2
Relative cell growth
Days
B
SPC-A1+CTL siRNA
SPC-A1+PKCε siRNA1
SPC-A1+PKCε siRNA2
Relative cell growth
Days
C
H292+CTL siRNA
H292+PKCε siRNA1
H292+PKCε siRNA2
Relative cell growth
Days
D
H1299+CTL siRNA
H1299+PKCε siRNA1
H1299+PKCε siRNA2
Relative cell growth
Days
E
H1299+CTL siRNA
H1299+PKCε siRNA1
H1299+PKCε siRNA2
F
CTL siRNA
PKCε siRNA1
PKCε siRNA2
Relative glutamate level
G
CTL siRNA
PKCε siRNA1
PKCε siRNA2
Relative glutamine level

Supplementary information, Figure S10. Knocking down PKCε inhibits the growth and glutamine metabolism of NSCLC cells. (A-D) NSCLC cell lines A549 (A), SPC-A1 (B), H292 (C), and H1299 (D) were transiently transfected with either control siRNA (CTL siRNA) or PKCε siRNAs. 24 hours later, the cells were seeded in 24-well plates at 3000 cells per well in 0.5 ml medium with 10% FBS. At the indicated times, cells were fixed in 3.7% formaldehyde and stained with 0.1% crystal violet. Dye was extracted with 10% acetic acid and the relative proliferation was determined by the absorbance at 595nm. Data represent the average of three independent experiments (mean±SD). *** P<0.001. **(E)** H1299 cells were transiently transfected with either control siRNA (CTL siRNA) or PKCε siRNAs. 24 hours later, the cells were seeded in 6-well plate at 500 cells per well in 2 ml medium with 10% FBS. 10 days later, cells were fixed in 3.7% formaldehyde and stained with 0.1% crystal violet. **(F and G)** H1299 cells were transiently transfected with either control siRNA (CTL siRNA) or PKCε siRNAs. 48 hours later, the cells were collected and glutamate (F) and glutamine (G) were determined by different kits. Data represent the average of three independent experiments (mean±SD). * P<0.05, ** P<0.01.
