# Supplementary material for: Phosphorylation of glutaminase by PKCε is essential for its enzymatic activity and critically contributes to tumorigenesis
Source: Cell Res. 2018 Mar 7;28(6):655–69. doi: 10.1038/s41422-018-0021-y (PMC5993826; doi:10.1038/s41422-018-0021-y)
Supplement: Supplementary file 11 — Figure S11 [file 41422_2018_21_MOESM11_ESM.pdf]

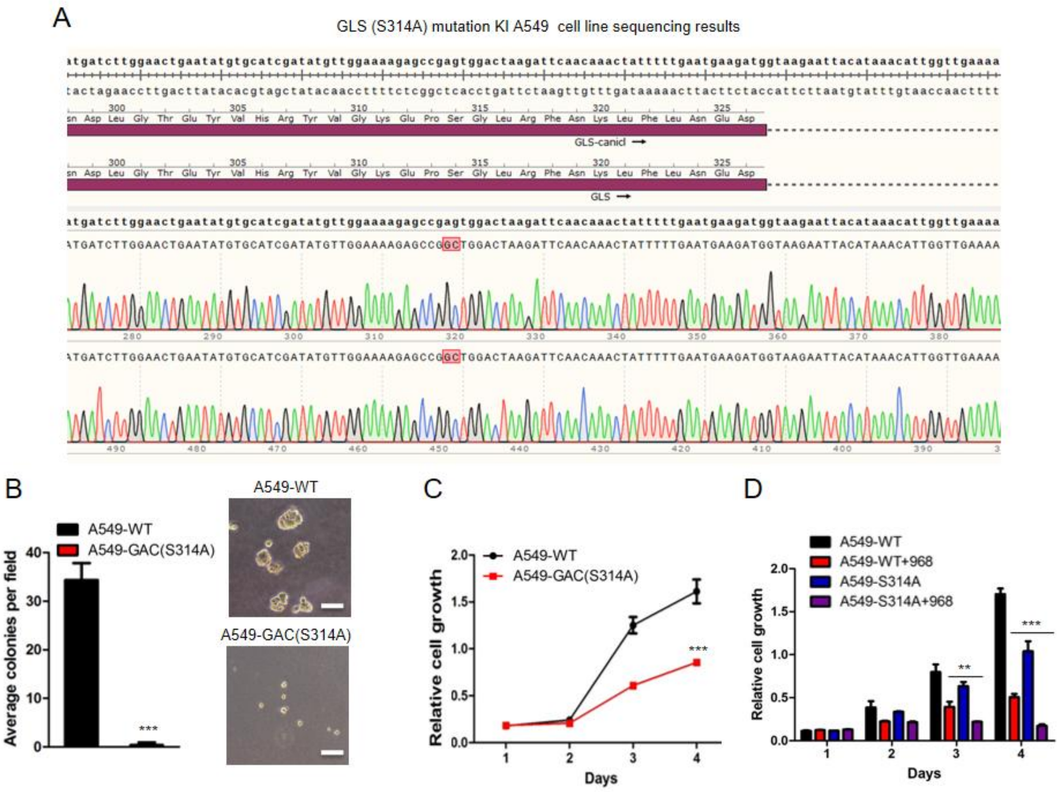
A
GLS (S314A) mutation KI A549 cell line sequencing results
GLS-canicl →
GLS →
B
A549-WT
A549-GAC(S314A)
Average colonies per field
***
C
A549-WT
A549-GAC(S314A)
Relative cell growth
Days
***
D
A549-WT
A549-WT+968
A549-S314A
A549-S314A+968
Relative cell growth
Days
**
***

Supplementary information, Figure S11. S11 Inhibition of GAC phosphorylation at S314 reverses the transformed phenotypes of A549 cells. (A) Schematic depiction of the sequencing results for GLS (S314A) mutation in A549 cells. **(B)** Soft agar assay. A549-WT cells or A549-GAC(S314A) stable cells were suspended with RPMI 1640 supplemented with 10% FBS and 0.3% agarose followed by plating on top of a solidified layer of RPMI 1640 supplemented with 10% FBS and 0.5% agarose. Two weeks later, colonies larger than 50μM were scored. Data represent the average of three independent experiments (mean±SD). *** *P*<0.001 (Left figure). The photographs of colonies in soft agar assay. Scale bars: 100μm. (Right figure). **(C)** A549-WT and A549-GAC(S314A) cells were cultured in RPMI 1640 with 10% FBS for indicated times, and then cells were fixed in 3.7% formaldehyde and stained with 0.1% crystal violet. Dye was extracted with 10% acetic acid and the relative proliferation was determined by the absorbance at 595nm. Data represent the average of three independent experiments (mean±SD). ****P*<0.001. **(D)** A549-WT cells and A549-GAC (S314A) cells were treated with or without 10μM 968 for the indicated times, and then cells were fixed in 3.7% formaldehyde and stained with 0.1% crystal violet. Dye was extracted with 10% acetic acid and the relative proliferation was determined by the absorbance at 595nm. Data represent the average of three independent experiments (mean±SD). ***P*<0.01, ****P*<0.001.
