# Supplementary material for: Phosphorylation of glutaminase by PKCε is essential for its enzymatic activity and critically contributes to tumorigenesis
Source: Cell Res. 2018 Mar 7;28(6):655–69. doi: 10.1038/s41422-018-0021-y (PMC5993826; doi:10.1038/s41422-018-0021-y)
Supplement: Supplementary file 12 — Figure S12 [file 41422_2018_21_MOESM12_ESM.pdf]

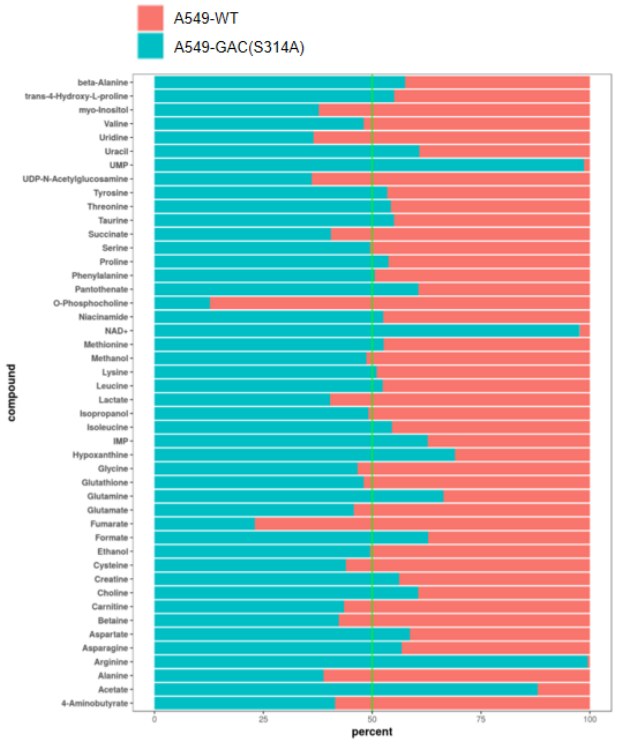

A549-WT
A549-GAC(S314A)
beta-Alanine
trans-4-Hydroxy-L-proline
myo-Inositol
Valine
Uridine
Uracil
UMP
UDP-N-Acetylglucosamine
Tyrosine
Threonine
Taurine
Succinate
Serine
Proline
Phenylalanine
Pantothenate
O-Phosphocholine
Niacinamide
NAD+
Methionine
Methanol
Lysine
Leucine
Lactate
Isopropanol
Isoleucine
IMP
Hypoxanthine
Glycine
Glutathione
Glutamine
Glutamate
Fumarate
Formate
Ethanol
Cysteine
Creatine
Choline
Carnitine
Betaine
Aspartate
Asparagine
Arginine
Alanine
Acetate
4-Aminobutyrate
compound
0
25
50
75
100
percent

**Supplementary information, Figure S12. The metabolomics analysis in A549-WT cells and A549-GAC (S314A) cells.** The percentage of different metabolites between A549-WT cells (red) and A549-GAC (S314A) cells (green).
