# Supplementary material for: Phosphorylation of glutaminase by PKCε is essential for its enzymatic activity and critically contributes to tumorigenesis
Source: Cell Res. 2018 Mar 7;28(6):655–69. doi: 10.1038/s41422-018-0021-y (PMC5993826; doi:10.1038/s41422-018-0021-y)
Supplement: Supplementary file 13 — Supplementary table 1 [file 41422_2018_21_MOESM13_ESM.pdf]

## A. The mass spectrometry analysis of phosphorylation sites in HBE cell

| Phosphorylation sites | Peptide sequence |
|---|---|
| T170 | RT#SDPRL |
| S171 | RTS#DPRL |
| T185 | KECMDMLRLT#LQTTSDGVMLDKD |
| S242 | KKQS#GGKV |
| S380 | KMAGNEYVGFSNATFQS#ERE |
| S593 | RMES#LGERS |
| | |

## B. The mass spectrometry analysis of phosphorylation sites in H1299 cell

| Phosphorylation sites | Peptide sequence |
|---|---|
| T112 | KDSPGET#DAFGNSEGKEMVAAGDNKIKQ |
| S171 | RTS#DPRL |
| T185 | RLT#LQTTSDGVMLDKD |
| T188 | RLTLQT#TSDGVMLDKD |
| S274 | RHS#IGDTKV |
| T278 | RHSIGDT#KV |
| S314 | KEPS#GLRFNKL |
| S511 | KMGNS#VKG |
| S576 | KKVS#PESSDDTSTTVVYRMESLGERS |
| S593 | RMES#LGERS |
| | |

## C. Comparing the phosphorylation sites in H1299 with HBE cells

| HBE | H1299 |
|---|---|
| T170 | T112 |
| S171 | S171 |
| T185 | T185 |
| S242 | T188 |
| S380 | S274 |
| S593 | T278 |
| | S314 |
| | S511 |
| | S576 |
| | S593 |

**Table S1.** The GAC phosphorylation sites in HBE cells and H1299 cells. **(A)** The mass spectrometry analysis of GAC phosphorylation sites in HBE cells. **(B)** The mass spectrometry analysis of GAC phosphorylation sites in H1299 cells. **(C)** Comparing the GAC phosphorylation sites in H1299 cells with those in HBE cells from the mass spectrometry analysis results.
