# Supplementary figures and images for: Phosphorylation of glutaminase by PKCε is essential for its enzymatic activity and critically contributes to tumorigenesis
Source: Cell Res. 2018 Mar 7;28(6):655–69. doi: 10.1038/s41422-018-0021-y (PMC5993826; doi:10.1038/s41422-018-0021-y)

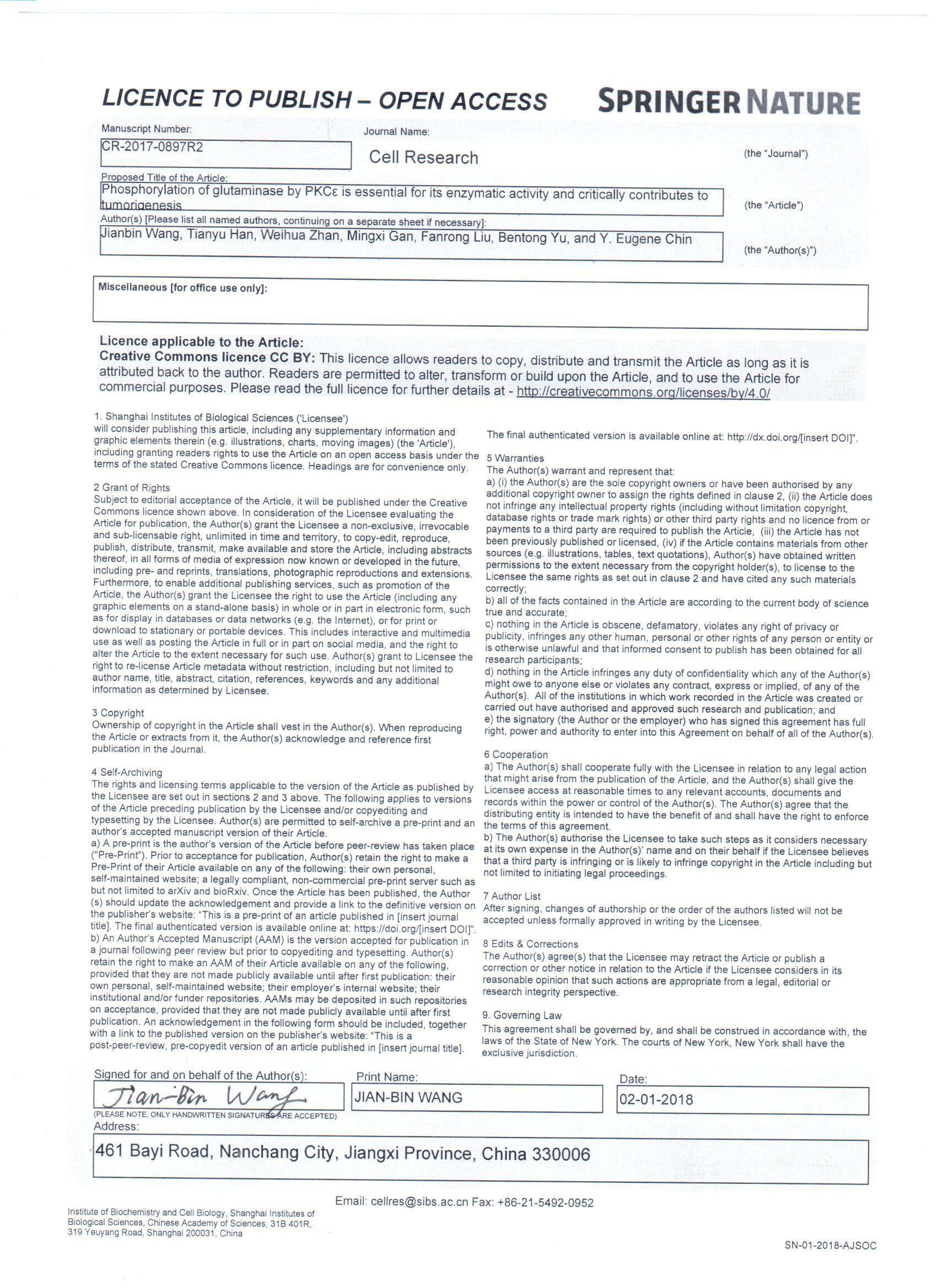

Supplement: Supplementary file 24 — licence [file 41422_2018_21_MOESM24_ESM.tif]
